# Beneficial Effects of Resveratrol and γ-Cyclodextrin on the Hematological and Biochemical Parameters of Healthy Wistar Rats Treated with Cisplatin: A PCA Approach

**DOI:** 10.3390/biomedicines11102726

**Published:** 2023-10-08

**Authors:** Nicoleta-Gabriela Hădărugă, Zeno Gârban, Cornel Baltă, Florin Muselin, Daniel-Ioan Hădărugă, Mircea Riviş

**Affiliations:** 1 Department of Food Science, University of Life Sciences “King Mihai I” from Timisoara, Calea Aradului 119, 300645 Timisoara, Romania; 2 Working Group for Xenobiochemistry, Romanian Academy—Timisoara Branch, Mihai Viteazu Bd. 24, 300223 Timisoara, Romania; zeno.garban@yahoo.com; 3 “Aurel Ardelean” Institute of Life Sciences, “Vasile Goldis” Western University, Liviu Rebreanu 86, 310414 Arad, Romania; balta.cornel@uvvg.ro; 4 Department of Toxicology and Toxicoses, Plant Biology and Medicinal Plants, University of Life Sciences “King Mihai I” from Timisoara, Calea Aradului 119, 300645 Timisoara, Romania; florinmuselin@usvt.ro; 5 Department of Applied Chemistry, Organic and Natural Compounds Engineering, Polytechnic University of Timisoara, Carol Telbisz 6, 300001 Timisoara, Romania; daniel.hadaruga@upt.ro; 6 Department of Anesthesiology and Oral Surgery, “Victor Babes” University of Medicine and Pharmacy Timisoara, Eftimie Murgu Sq. 2, 300041 Timisoara, Romania; mrivis@yahoo.com

**Keywords:** cisplatin, anticancer drugs, γ-cyclodextrin, resveratrol, hematological and biochemical parameters, side effects, Wistar rats, principal component analysis

## Abstract

It is well known that platinum-based antineoplastic agents, including cisplatin (CP), have side effects that limit their use. Nefrotoxicity, neurotoxicity, and hemolytic anemia are the most common side effects. There are few studies on the reduction in these effects that involves nanoencapsulation; however, almost none involve cyclodextrins (CDs). Changes in the hematological and biochemical parameters of healthy Wistar rats treated with solutions of γ-cyclodextrin/resveratrol/cisplatin (γ-CD/Rv/CP) ternary complexes are investigated for the first time. They are intraperitoneally injected with γ-CD/Rv/CP solutions containing 5 mg CP/kg.b.w. Single shots were administered to six groups of Wistar rats (six individuals for every group) using γ-CD/Rv/CP, γ-CD/CP, γ-CD/Rv complexes, as well as positive- and negative-control groups, respectively. Thirty-two hematological and biochemical parameters were evaluated from blood samples and used as input variables for the principal component analysis (PCA) discrimination of the groups. The best protection was obtained for the γ-CD/Rv/CP ternary complex, which determined closer biochemical values to the control group. These values significantly differ from those of the γ-CD/CP treated group, especially for the IP, UA, and T-Pro kidney-related biochemical parameters. This finding proves the beneficial influence of Rv during CP administration through CD-based carriers.

## 1. Introduction

Cancer is one of the most widely spread diseases affecting both young/children and adult/old people. There are many types of cancer, including lung, prostate, breast, colorectal, and stomach [1]. Depending on the cancer type, many antineoplastic agents and protocols are used. According to the Anatomical Therapeutic Chemical (ATC) Classification, there are four subclasses of antineoplastic and immunomodulation agents, while the antineoplastic agents are also subdivided into seven groups [2]. They are alkylating agents, antimetabolites, plant alkaloids and other natural products, cytotoxic antibiotics and related substances, protein kinase inhibitors, monoclonal antibodies and antibody drug conjugates, and other antineoplastic agents. Many of these antineoplastic agents are highly toxic and harmful to the human body, especially alkylating agents, alkaloids, inorganic, and organometallic compounds. The latter includes platinum compounds, such as cisplatin, carboplatin, or oxaliplatin. Other organometallic compounds investigated for their antineoplastic properties belong to metallocene, metal-based half-sandwich complexes, organometallic carbene–, carbonyl–, or π–ligand complexes [3]. Not only platinum, but iron, titanium, palladium, ruthenium, osmium, iridium, rhodium, and rhenium-based (organo)metallic compounds, and other miscellaneous compounds were studied as possible antineoplastic agents [4,5].

Cisplatin (CP), *cis*-diamminedichloroplatinum(II), has been used as an antineoplastic agent for more than fifty years, revealing good antineoplastic activity against ovarian and testicular cancers; head, neck, and cervical cancers; various lung cancers; brain tumors; or neuroblastomas [6]. However, there are many harmful side effects on renal, nervous, and otic systems [7,8,9,10,11]. The amelioration of these effects was extensively studied through combined treatments using various natural compounds and systems, or through the administration of CP using drug carriers. The adjuvant chemotherapy of HSS (a protein extracted from *Tegillarca granosa* L.) with CP and docetaxel against non-small-cell lung cancer was evaluated from the toxicity point of view. It was observed that adjuvant chemotherapy could reduce the toxicity of docetaxel–CP treatment by increasing the numbers of WBC and PLT, decreasing the levels of ALT, AST, and BUN, as well as by the improvement of body weight and food consumption [12]. A combination of herbo-mineral extracts based on Maharasanadi qoath, *Tinospora cordifolia*, *Rubia cordifolia*, *Emblica officinalis*, *Moringa pterigosperma*, *Glycyrrhiza glabra*, and powders of *Balsamodendron mukul* and Shankha bhasma, known as Septilin, was investigated for its beneficial effect on the reduction in the CP-induced toxicity in Swiss albino mice. It was found that Septilin supplementation could reduce the toxicity of CP in liver, somatic, and bone marrow cells [10]. The toxicity of CP during the chronic treatment of the rats was studied after the administration of bixin and annatto seeds through the food. The nephrotoxicity induced with a single intraperitoneal administration revealed a reduced level of neutrophil counts in the bixin-based group. Renal injury was also attenuated in this case [11]. In another study, the acute toxicity of CP was evaluated for pH-sensitive liposome carriers in mice. The LD_50_ values were approximately three-times higher for the CP-loaded liposome systems, in comparison with the control group. Additionally, the hematological and biochemical parameters were slightly altered after the administration of the CP-loaded liposomes, proving the protection against CP-induced toxicity after an intraperitoneal administration [9]. In a randomized, double-blind, placebo-controlled study, the supplementation with antioxidant micronutrients (vitamins C, E, and selenium) in cancer patients treated with CP revealed a significantly reduced loss of high-tone hearing. A high correlation between the reduced/oxidized vitamin C ratio and malondialdehyde, markers of oxidative stress, and CP-induced ototoxicity and nephrotoxicity was observed [13].

Cyclodextrins (CDs) are appropriate hosts for delivering various antineoplastic agents having geometrically compatible and mostly hydrophobic molecules. Natural β- and γ-CD, as well as semi-synthetically modified CDs (e.g., 2-hydroxypropyl-β-CD), were studied/applied for delivering these drugs. The enhancement of the apparent water solubility and bioavailability/bioaccessibility was obtained through β-CD/doxorubicin, HP-β-CD/melphalan, or HP-β-CD/CP complexations [14]. More complex delivery supramolecular systems based on CDs, such as liposomes, niosomes, nanosponges, micelles, polymeric millirods, nanoparticles, magnetic nanoparticles, CD-grafted polymeric nanocarriers, short-interfering RNA delivering systems, monoclonal antibody drug conjugates, or supramolecular vesicles were studied [14,15,16,17]. Organometallic anticancer drugs were studied for the efficiency of molecular encapsulation and delivery through CD complexations. Oxaliplatin/α-, β-, and HP-β-CD complexes or oxaliplatin-based β-CD/phillipsite composites were studied as therapies against HCT116 and MCF-7 cells for colorectal cancer. Methyl-β-CD/carboplatin and methyl-β-CD/5-fluorouracil were investigated for their effects against human breast cancer cells, while HP-α-CD/carboplatin was studied for its effects against brain tumors [18,19,20,21]. On the other hand, oxyresveratrol and resveratrol (Rv) complexes with CD-based nanosponges presented enhanced bioactivity against prostate (PC-3) and colon (HT-29 and HCT-116) cancer cell lines [22]. In similar studies, sulfobutylether-β-CD/Rv was studied against a human breast cancer cell line (MCF-7), while Rv-in-CD-in-liposome systems were investigated for their in vitro cytotoxicity against HT-29 colon cancer cell lines with enhanced delivery properties [23,24]. CD/CP complexes were also studied as anticancer agents with enhanced properties. Tumor inhibition was studied by an *Agrobacterium tumefaciens*-induced potato disc tumor assay for CP and HP-β-CD/CP or HP-β-CD-based gelatin nanoparticles, the highest activity being observed for HP-β-CD/CP complexes [25]. Targeted therapy was investigated for a slow and controlled release system to deliver CP using magnetogel nanospheres composed of CP-loaded alginate/β-CD [26]. Similar CP delivery systems were obtained for photoacoustic imaging-guided chemo-photothermal cancer therapy. β-CD/CP-loaded polydopamine nanoparticles via supramolecular self-assembly revealed highly potent in vitro anticancer activity against osteosarcoma 143B cells [27]. Other CP and CD-based supramolecular systems, such as nanovesicles, CD-capped gold nanoparticles, or hydrogels were studied as antineoplastic agents with good results [28,29,30,31].

The goal of this study is the evaluation of the changes in the hematological and biochemical parameters of healthy Wistar rats treated with solutions of γ-CD/Rv/CP ternary complexes in comparison to the γ-CD/Rv and γ-CD/CP binary complex control groups. This study on such ternary complexes is performed for the first time and reveals the beneficial effects of both Rv as an “on-site” antioxidant and γ-CD as a water solubility and controlled delivery enhancer, as is demonstrated by the principal component analysis (PCA) of kidney-related biochemical parameters.

## 2. Materials and Methods

### 2.1. Animals

Wistar rats were purchased from the Biobase of the “Iuliu Haţieganu” University of Medicine and Pharmacy from Cluj-Napoca (Romania). The 8-week-old male and female rats were acclimated (light/dark cycles of 12/12 h, temperature of 20–25 °C, and relative humidity ~ 50%) at least 2 weeks before the experiments. Stainless-steel cages, type 1290 (SC Sapaco 2000 SA, Bucharest, Romania), with dimensions of L × l × H = 425 × 266 × 155 mm^3^, total bottom area of 820 cm^2^, and 24 cages/unit were used. The cages were tapped daily with autoclaved bedding (“Chipsi-60L”, Animax, Timişoara, Romania) and the animals had free access to tap water and sterilized food (Agroland Bussiness System SRL, Timişoara, Romania). The handling and experiments on the Wistar rats respected the animal study protocol that was approved by the Institutional Review Board of the Banat’s University of Agricultural Sciences and Veterinary Medicine “King Mihai I of Romania” from Timişoara (Romania), with the protocol code related to the research grant “PCCE_140_Act_ad_2” and registration No 10679/30.11.2011 for studies involving animals for the “In vivo testing of compounds and analysis”.

### 2.2. Materials, Drugs, and Hematological and Biochemical Kits

γ-Cyclodextrin hydrate had a purity of >98%, a water content of 9.0% (loss during drying), and a residual oligosaccharide content < 0.5% (by HPLC), which included α- and β-CD (CycloLab R&D. Ltd., Budapest, Hungary). High-purity resveratrol of >99% was obtained from Sigma-Aldrich (St. Louis, MO, USA) and was HPLC grade. Saline solution 0.9% (pharmaceutical grade) and DMSO (pro-analysis grade, Fluka/Sigma-Aldrich, St. Louis, MO, USA) were used for the preparation of the drug solutions. A CP solution of the analyzed drug (cisplatin content of 50 mg/100 mL, EBEWE Pharma Ges.m.b.H. Nfg.KG, Unterach, Austria) was used for preparing CP-based samples. The hematological and biochemical kits (for WBC, LYM, MID, GRA, LY%, MI%, GR%, RBC, HGB, HCT, MCV, MCH, MCHC, RDWc, PLT, PCT, MPV, PDWc, as well as GOT/AST; GPT/ALT; GGT; total bilirubin, T-Bil; BUN; UA; creatinine, Cre; total protein, T-Pro; albumin, Alb; CPK; calcium, Ca; magnesium, Mg; and inorganic phosphorus, IP) were obtained from Arkray Inc. (Kyoto, Japan).

### 2.3. Preparation of γ-CD/Rv/CP Ternary Complexes and γ-CD/Rv and γ-CD/CP Binary Complexes in Solution

γ-CD/Rv/CP ternary complexes and γ-CD/Rv and γ-CD/CP binary complexes were prepared in solution, at a final saline solution:DMSO volume ratio of 1:1. The negative-control solution (code “C” for PCA analysis) consisted of 10 mL of 0.9% saline solution and 10 mL of DMSO, while the positive-control solutions were obtained by dissolving 47.5 mg of γ-CD hydrate (0.033 mmol) in a 20 mL saline solution:DMSO mixture (code “G”), respectively, by diluting 10 mL of the standard CP solution (concentration of 1 mg/mL, 0.017 mmol) with 10 mL of DMSO (code “P”). The ternary and binary complexes were obtained as follows: 47.5 mg γ-CD hydrate and 10 mL of standard CP solution were dissolved in 10 mL of DMSO (γ-CD:CP molar ratio of 2:1, code “PG”), 47.5 mg γ-CD hydrate and 7.6 mg Rv were dissolved in a 20 mL saline solution:DMSO mixture (γ-CD:Rv molar ratio of 2:2, code “RG”), and, finally, 47.5 mg γ-CD hydrate, 7.6 mg Rv, and 10 mL of standard CP solution were dissolved in 10 mL of DMSO (γ-CD:Rv:CP molar ratio of 2:2:1, code “RP” for PCA). All control and sample solutions were stored for 24 h at 4 °C in order to reach the host–guest association–dissociation equilibrium for the binary and ternary complexes.

### 2.4. Experimental Design

Six groups corresponding to the abovementioned negative- and positive-control solutions, as well as ternary and binary complex solutions consisting of six male and female Wistar rats, were prepared. A single injection of solution was administered intraperitoneally. According to every rat weight, volumes of 1.72–3.41 mL of control and γ-CD-based solutions (that corresponded to the same dose of 5 mg CP/kg.b.w. for samples containing CP) were used (see Table 1). Blood samples were collected from vena cava on the 6th day after the sample administration (under isoflurane anesthesia, oxygen flow of 2 L/min, and isoflurane administration of 5 mL/min for induction, followed by 1.5 mL/min), followed by euthanasia.

### 2.5. Hematological and Biochemical Investigations

Thirty-two hematological and biochemical parameters were considered. The hematological parameters were determined after blood collection using a multiparametric hematology analyzer (Abacus Junior Vet 2.85, Diatron Messtechnik GmbH, Wien, Austria). The following hematological parameters were determined: WBC (white blood cells, expressed as 10^9^/L), LYM (lymphocytes, 10^9^/L), MID (middle cells, 10^9^/L), GRA (granulocytes, 10^9^/L), LY% (lymphocyte, %), MI% (middle cell, %), GR% (granulocyte, %), RBC (red blood cells, 10^12^/L), HGB (hemoglobin, g/dL), HCT (hematocrit, %), MCV (mean corpuscular volume, fL), MCH (mean corpuscular hemoglobin, pg), MCHC (mean corpuscular hemoglobin concentration, g/dL), RDWc (red blood cell distribution Width, %), PLT (platelet/thrombocytes, 10^9^/L), PCT (plateletcrit, %), MPV (mean platelet volume, fL), and PDWc (platelet distribution width, %). Biochemical determinations were performed on heparinized blood plasma samples (the blood sample was placed in a 2 mL microcentrifuge tube and centrifuged at 2000 rpm for 5 min using a MicroONE microcentrifuge, Tomy Digital Biology, Co., Ltd., Tokyo, Japan). Liver-related biochemical parameters were GOT/AST (glutamic-oxalacetic transaminase/aspartate transaminase, expressed as IU/L), GPT/ALT (glutamic-pyruvic transaminase/alanine transaminase, IU/L), GGT (gamma-glutamyl transferase, IU/L), and T-Bil (total bilirubin, mg/dL). For the kidney, the following biochemical parameters were determined: BUN (blood urea nitrogen, expressed as mg/dL), UA (uric acid, mg/dL), Cre (creatinine, mg/dL), T-Pro (total protein, g/dL), Alb (albumin, g/dL), ALP (alkaline phosphatase, IU/L), and CPK (creatine phosphokinase, IU/L). Finally, calcium (Ca, mg/dL), magnesium (Mg, mg/dL), and inorganic phosphorus (IP, mg/dL) were also determined.

### 2.6. Molecular Modeling and γ-CD Docking of Resveratrol and Cisplatin

The geometrical compatibility and the favorable interaction energy of guest compounds (Rv and CP) with the host molecule, γ-CD, were theoretically evaluated by molecular modeling and docking experiments [32,33,34,35]. First, the molecules were built up using the Add Hydrogen & Model Build module in HyperChem 7.52 (HyperCube, Inc., Gainesville, FL, USA). The most stable conformations were obtained using the molecular mechanics MM+/Conformational Search module in the same package. The MM+ algorithm was Polak-Ribiere (conjugate gradient). All flexible bonds in γ-CD and Rv were considered in the conformational analysis, taking into account a range for the acyclic torsion variation of ±60° to ±180° and up to 8 simultaneous variations. Additionally, pyran rings in γ-CD were considered as flexible, with a range for the ring torsion flexing between ±30° and ±120°. The acceptance energy criterion was set to 4 kcal/mol for all molecules and usage directed method for choosing the initial conformations set to vary. Optimization termination was set at an RMS gradient of 0.01 kcal/mol. The minimum energy conformation (determined in vacuum conditions) for every molecule was selected for the docking experiments.

The docking of CP and Rv (separately or in competition) in the γ-CD cavity was performed with the same MM+ program by a geometry optimization of the binary and ternary complexes. The termination condition was the RMS gradient, which was set at 0.1 kcal/mol. The absolute energies of both minimum energy conformations and stable complexes were recorded and used for the determination of the host–guest interaction energy. It was calculated as the difference between the sum of minimum energy conformations of individual molecules and the absolute energy of the complex. A positive interaction energy revealed a stable complex.

### 2.7. Data and PCA Multivariate Statistical Analyses

All values for the hematological and biochemical parameters were presented as means ± standard deviations (SD, *n* = 6). One-way ANOVA and Basic Statistics & Tables modules in Statistica 7.1 software (StatSoft, Inc., Tulsa, OK, USA) were used. The significant differences between the parameter values were determined using Tukey’s HSD test (honestly significant difference) included in the One-way ANOVA module. Generally, the *p*-level for a significant difference was set at *p* < 0.05.

PCA is a powerful multivariate statistical analysis technique that allows us to discriminate objects. It only needs independent variables, X_1_, X_2_, …, X_n_ (no matter if they are correlated/partially correlated or not). These X variables are processed by PCA in such a way to provide PC variables (principal components or factors) using the matrix algebra. The new PCs were obtained using the following restrictions: maximum variance of the X data and orthogonality between PCs. The advantage of the PCA was the considerable reduction in the number of important variables (PCs) in comparison with the number of starting variables (X_1_ to X_n_). Almost all the time, the first two or three new variables (PC_1_ to PC_2_/PC_3_) are sufficient for explaining the variance in the data and finding the similarities/dissimilarities between the objects (cases). In this study, the hematological and biochemical parameters were considered for PCA. PCA was performed with the Unscrambler 6 (Camo, Trondheim, Norway). Eighteen hematological and fourteen biochemical parameters involved in the analyses of the blood and plasma samples from Wistar rats treated with control solutions, CP, and Rv binary and ternary γ-CD complexes were considered at the first step. After the step-by-step selection of the most important parameters, eight biochemical parameters provided a clear discrimination of the γ-CD/Rv/CP ternary complex and control groups against the non-antioxidant-protected γ-CD/CP binary complex group (see Section 3 and Section 4). The cross-validation method and centered data were selected for the PCA analysis.

## 3. Results

### 3.1. Hematological Parameters

**Figure 1:**
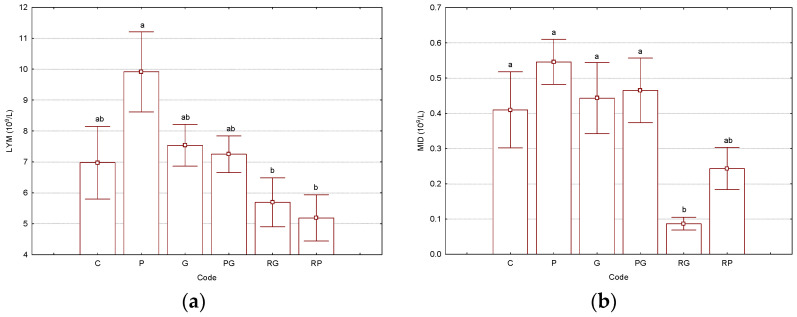
Results for the selected hematological parameters LYMs—lymphocytes, 10^9^/L (**a**) and MIDs—middle cells, 10^9^/L (**b**), determined from the Wistar rat blood samples (“C”—control group, “P”—group treated with cisplatin, “G”—group treated with “empty” γ-cyclodextrin, “PG”—group treated with γ-cyclodextrin/cisplatin binary complex, “RG”—group treated with γ-cyclodextrin/resveratrol binary complex, “RP”—group treated with γ-cyclodextrin/resveratrol/cisplatin ternary complex). Bars with different letters are significantly different, according to the Tukey HSD test (*p* < 0.05). All *p*-values are presented in the Supplementary Materials file (Tables S5–S22). The number of replicate determinations was *n* = 6. The “Whisker”-type representation with average values and error bars (±0.95·standard error) is used.

The treatment of Wistar rats with the CP solution modified some of the hematological parameter values, especially those related to the red blood cell counts. Even the RBC, HGB, and HCT had values close to the lower limits of the normal ranges for the “P” group; they did not significantly differ from the other groups. However, many hematological parameters were slightly affected by CP administration (single injection and analysis at the 6th day). Values of the representative hematological parameters are presented in Figure 1. All results related to the hematological parameters can be found in the Supplementary Materials file (Table S1). The WBC values were in the range of 8.1–11.3 × 10^9^/L, with lower values for “RG” and “RP” binary and ternary complexes. However, the SD values were relatively high, while *p* had the lowest values of 0.15 for these cases (see Supplementary Materials file, Table S5). Lymphocytes and middle-cell counts were significantly different (*p* < 0.05) among “P” and “RG” treatments. For LYM, the lowest values were observed for all γ-CD-based treatments, especially for “RG” and “RP” cases, most probably due to the molecular encapsulation capability of γ-CD for other components “in vivo”, such as phospholipids, glycolipids, and sterols from the cell membranes (Figure 1a). For the MID values, the “RG” case was significantly different (*p* < 0.05) from all the others. Additionally, the MID value for the ternary complex “RP” was 2.5-times higher than the “RG” case; however, it was half the value in comparison with all the other cases (Figure 1b). Granulocyte counts had no significant variations. However, the lowest value was obtained for the “P” treatment. Additionally, the γ-CD-based treatments provided slightly lower values than the control “C”. The percent distribution of these cells followed their counts, but without significant variations. LY%, MI%, and GR% were in the ranges of 63.3–75.1, 1.1–4.9, and 20.0–33.3%, respectively (see Supplementary Materials file, Tables S1 and S6–S11, where the normal values were also included).

Red blood cell count, hemoglobin, and hematocrit had lower values for the case of Wistar rats treated with the CP solution (code “P”). RBC counts had values of 7.1 × 10^12^/L for the “P” case and 7.9–8.7 × 10^12^/L for the others. The highest values were observed for control-group “C” and the group treated with “empty” γ-CD (code “G”). For HGB and HCT, the lowest values were 12.3 g/dL and 35.95% in the case of group “P”. However, differences were not statistically significant (see Supplementary Materials file, Tables S12–S14). Almost no variations in the values for the mean corpuscular volume (MCV), mean corpuscular hemoglobin (MCH), and the corresponding concentration (MCHC) were observed. These were in the ranges of 48.0–50.3 fL, 16.7–17.5 pg, and 33.2–35.2 g/dL, respectively (see Supplementary Materials file, Tables S1 and S15–S17). The maximum value for the red blood cell distribution width was determined for control-group “C” (RDWc = 18.3%) and the lowest for the CP-treated group (code “P”, RDWc = 16.4%). However, the *p*-value was not significant (see Supplementary Materials file, Tables S1 and S18). Similar observations were made for the platelet/thrombocyte counts (PLTs) and plateletcrit (PCT) that had the lowest values for the “P” group (PLT = 663 × 10^9^/L and PCT = 0.39%) and the highest ones for the “RG” binary complex (PLT = 947 × 10^9^/L and PCT = 0.58%). No significant variations were observed for the mean platelet volume and platelet distribution width (5.7–6.1 fL and 29.1–31.1%, respectively; see Supplementary Materials file, Tables S1 and S18–S22, where the normal values were also included).

### 3.2. Biochemical Parameters

**Figure 2:**
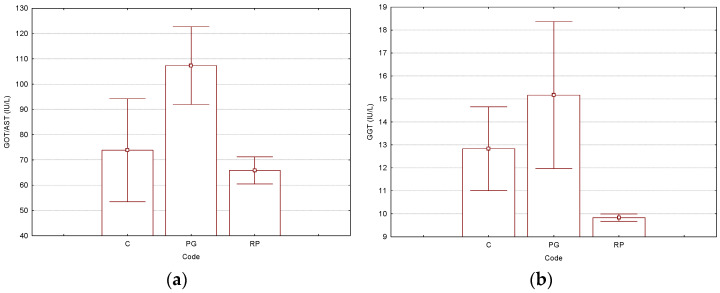
Results for the selected liver-related parameters GOT/AST (glutamic-oxalacetic transaminase/aspartate transaminase, IU/L) (**a**) and GGT (gamma-glutamyl transferase, IU/L) (**b**), determined from the Wistar rat plasma samples (“C”—control group, “PG”—group treated with γ-cyclodextrin/cisplatin binary complex, “RP”—group treated with γ-cyclodextrin/resveratrol/cisplatin ternary complex). The Tukey HSD test (*p* < 0.05) was used for the evaluation of the statistical significances (no statistically significant differences were observed). All *p*-values are presented in the Supplementary Materials file (Tables S23–S26). The number of replicate determinations was *n* = 6. The “Whisker”-type representation with average values and error bars (±0.95·standard error) is used.

For the biochemical parameters, the studies focused on the influence of γ-CD molecular encapsulation. Both the γ-CD/CP binary complex (code “PG”) and γ-CD/Rv/CP ternary complex (code “RP”) were compared with control-group “C”. Liver-related biochemical parameters had higher values for the group treated with the γ-CD/CP binary complex (code “PG”), in comparison with the antioxidant-based ternary complex case (code “RP”), which had values close to control-group “C”. However, *p*-values were not too low, such as in kidney-related biochemical parameters (see below). All the results for the liver-related biochemical parameters determined in Wistar rat plasma samples are presented in Figure 2 and the Supplementary Materials file (Tables S2 and S23–S26 for the Tukey HSD test, where the normal values were also included). The GOT/AST values were 1.5–1.6-times higher for the group treated with the γ-CD/CP binary complex (higher than the upper limit of the normal values in rats, see Supplementary Materials file, Table S2). These values presented similar results for the other liver-related biochemical parameters, i.e., GPT/ALT, GGT, and T-Bil. The binary complex “PG” had the following values: 68.2, 15.2, and 0.70 mg/dL, respectively (all close to the upper limits of the normal ranges). Control “C” and ternary complex “RP”-treated groups had 1.2–1.4-, 1.2–1.5-, and 1.8–2.1-times lower values, which were in the normal ranges (Figure 2 and Supplementary Materials file, Tables S2 and S23–S26 for the Tukey HSD test, where the normal values were also included).

**Figure 3:**
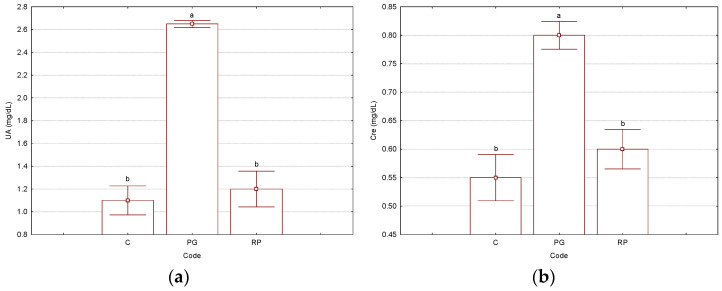
Results for the significant kidney-related parameters: UA—uric acid, mg/dL (**a**) and Cre—creatinine, mg/dL (**b**), determined from the Wistar rat plasma samples (“C”—control group, “PG”—group treated with γ-cyclodextrin/cisplatin binary complex, “RP”—group treated with γ-cyclodextrin/resveratrol/cisplatin ternary complex). Bars with different letters are significantly different, according to Tukey HSD test (*p* < 0.05). All *p*-values are presented in the Supplementary Materials file (Tables S27–S33). The number of replicate determinations was *n* = 6. The “Whisker”-type representation with average values and error bars (±0.95·standard error) is used.

Kidney-related biochemical parameters had values significantly different for the “PG” binary complex, in comparison with the antioxidant-protected “RP” ternary complex and control “C” groups. These latter groups presented similar values for almost all kidney-related biochemical parameters (except protein-based parameters). Thus, blood urea nitrogen and uric acid parameter values were 1.2–1.3- and 2.2–2.4-times lower (*p* < 2 × 10^−4^), respectively, in the Rv-based ternary complex and control groups, in comparison with the non-antioxidant protected binary complex used for the treatment. Additionally, the creatinine was 1.3–1.4-times lower in the control and antioxidant-protected treatment cases (*p* < 4 × 10^−3^). Total proteins and albumins were not significantly affected by the administration of the γ-CD/CP binary complex, because the values were very similar to the case of the group treated with the ternary complex and slightly lower for the control group. On the other hand, the enzyme alkaline phosphatase had values 1.2–1.5-times lower for the ternary complex and control groups; however, these differences were not statistically significant. No concluding influence of the non-protected binary complex treatment could be observed for the muscle tissue-related enzyme creatine phosphokinase. All experimental, normal, and *p*-values from the Tukey HSD test are presented in Figure 3 and the Supplementary Materials file (Tables S3 and S27–S33, where the normal values were also included).

Finally, calcium (Ca), magnesium (Mg), and inorganic phosphorus (IP) were evaluated in the non-antioxidant-protected γ-CD/CP binary complex group (“PG”), in comparison with the antioxidant-protected γ-CD/Rv/CP ternary complex and control groups (“RP” and “C”). For the “PG” case, all these biochemical parameter values were higher; however, only for Mg was a statistically significant difference observed (*p* < 0.01; see Supplementary Materials file, Tables S4 and S34–S36 for the Tukey HSD test results; the normal values were also included).

### 3.3. Principal Component Analysis of the Hematological and Biochemical Parameters

The attempt to analyze the data from all hematological parameter determinations using the PCA multivariate statistical analysis technique did not provide valuable classifications (see Supplementary Materials file, Figures S1–S6). These results suggest the comparison of non-antioxidant-protected and antioxidant-protected treatment groups, as well as the control group (“PG”, “RP”, and “C”, respectively). If all biochemical parameters were used as input variables for the PCA analysis, no clear classification was obtained. Even if the ALP, CPK, GOT/AST, and GPT/ALT variables were removed, we did not obtain better results (see Supplementary Materials file, Figures S7–S12).

Significant classifications were achieved by using T-Bil, UA, Cre, T-Pro, Alb, Ca, Mg, and IP as input variables for the PCA analysis. The non-antioxidant-protected γ-CD/CP binary complex cases were all classified in the top-right region of the PC_2_ versus PC_1_ scores plot, while the cases corresponding to the antioxidant-protected γ-CD/Rv/CP ternary complex and control groups were located on in the bottom-left side (without a clear discrimination between them). The most important variables for this classification were the total phosphorus and uric acid (IP and UA) variables for the PC_1_ and total protein and uric acid (T-Pro and UA) variables for PC_2_, as was clearly observed from the PC_2_ versus PC_1_ loadings plot. The first two PCs explain 81% of the variance of the data, while PC_3_ has an influence of only 8% (see Supplementary Materials file, Figures S15 and S16).

### 3.4. Molecular Modeling and Docking of Resveratrol and Cisplatin in γ-CD

**Figure 4:**
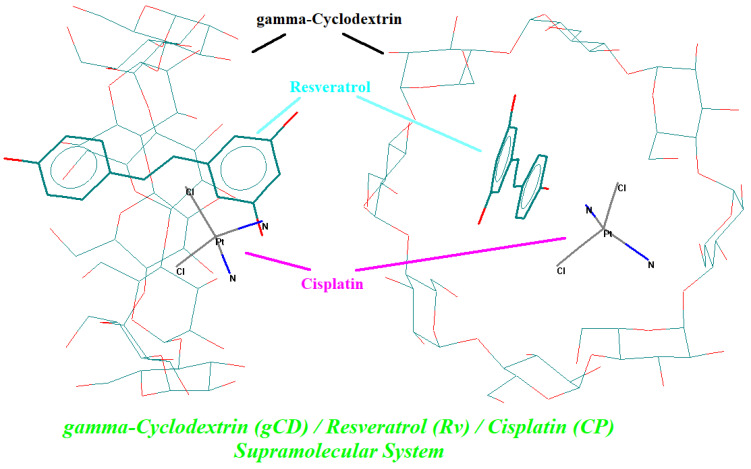
Molecular-modeled γ-cyclodextrin/resveratrol/cisplatin ternary complex at the minimum energy stage, obtained by MM+ geometry optimization; guest compounds are in bold (resveratrol—cyan; cisplatin—pink).

The possibility of formation of both binary and ternary complexes was proved by theoretical molecular modeling and docking experiments. First, the molecules were built up, molecularly modeled, and geometrically optimized using the molecular mechanics MM+ module in the HyperChem package. Then, all flexible bonds and rings (in γ-CD molecule) were selected for performing the conformational analysis. The minimum energy conformation of the molecule (the most stable conformation) in a vacuum was selected for the host–guest docking experiments. Various orientations of the host and guest molecules were considered, especially with the guest molecules oriented to the secondary face of γ-CD along the OZ axis. The best results were obtained when the starting optimization process had a distance between the γ-CD and CP gravity centers of ~5.5 Å. For the γ-CD/Rv binary complex, this starting distance was 10 Å; however, the closest OH group from the C4′ position was at 4.1 Å from the gravity center of the γ-CD, along the OZ axis. For the γ-CD/Rv/CP ternary complex, both guest molecules (Rv and CP) were oriented to the secondary face of γ-CD in the same way and at similar distances. After the geometry optimization of the binary and ternary complexes, the absolute energy of the stable complex was used for the evaluation of the host–guest interaction energy in a vacuum. All complexes revealed positive interaction energies, with the highest value for the γ-CD/Rv/CP ternary complex of 26.48 kcal/mol (units were arbitrary and could only be used for comparisons). The energy values for stable conformation, the optimized complex, and the host–guest interaction, as well as the molecular-modeled γ-CD/Rv/CP ternary complex in the most stable stage are presented in Table 2 and Figure 4, respectively [35] (see also the Supplementary Materials file, Figures S17 and S18, for the stable binary complexes).

## 4. Discussion

The beneficial and healthy effects of the presence of the antioxidant Rv co-encapsulated with CP in a γ-CD-based ternary complex were proved through the discrimination of the hematological and especially biochemical parameters for the intraperitoneally injected Wistar rats. Among PCA, various statistical parameters were used for the evaluation of the statistically significant differences between groups. First, the mean values and SDs were considered. The SD values for every multiplicate determination (number of cases, *n* = 6) were determined as the square root values of the sum of the squared differences between the actual and mean values of the observations, divided by the number of cases. Another classic statistical parameter was the standard error (SE, not presented in this study), which represented the standard deviation of the mean. The SE values could be determined as the square root values of the squared sample variances, divided by the number of cases. Both SD and SE were correlated and belonged to the descriptive statistics, which assumed the separate determination of the SD or SE values for each variable [36]. On the other hand, *p*-values (or *p*-level) expressed the classic statistical significance of a result and were based on an “a priori” null hypothesis significance [37]. Thus, a high *p*-value revealed a low probability to achieve a “valid” result (or a representative of a population). Generally, the standard *p*-value did not belong to the statistical inference. As a consequence, an alternative for the standard (classic) *p*-values were confidence intervals (CIs), statistical effects sizes (ESs), Bayes factors, and exploratory data analysis (EDA) [37,38]. In this study, the EDA methods were considered for the evaluation of the systematic relations between variables and groups. They were “post hoc” comparison methods and allowed for the “a posteriori” evaluation of the statistically significant differences between groups [39,40,41]. Thus, the groups in this study (both hematological and biochemical parameters) were compared by the “post hoc” Tukey HSD test, which is a highly recommended test when performing all pairwise comparisons. This is a powerful statistical tool used for the determination of the statistically significant relationship between two sets of data [42]. According to the Tukey HSD test, only some of the kidney-related biochemical parameters and the magnesium level had significantly different values (*p* < 0.05, UA and Cre, respectively, and Mg for “C” and “RP” groups against the “PG” group), in comparison with the hematological and other biochemical parameters (see explanations below and the Tukey HSD test results for all studied parameters in the Supplementary Materials file, Tables S5–S36).

Differences between the hematological parameter values exist, especially for the group treated with γ-CD/CP binary complex; however, the statistical parameters are less significant. This is probably due to the relatively reduced time range from the administration and blood analysis. The same observations resulted from other similar studies on mice and rats, where no significant changes in the body weight and hematological parameter values were observed after the administration of CP-based formulations at a 5 mg/kg body weight and after 6 days [11,12,27]. LY% was slightly higher than the normal limits in rats; however, the total LYM count was in the normal range [43,44,45,46]. No lymphocytopenia (low level of LYM) was observed after CP or CP-based formulation treatments. A decrease in the iron-related hematological parameters was observed for the γ-CD/CP-treated group. The RBC and HGB values were only 7.1 × 10^12^/L and 12.3 g/dL (slightly lower than the normal values of 5.3–10 × 10^12^/L and 14–18 g/dL, respectively) [43,44,45,46], whereas for the γ-CD/CP binary and γ-CD/Rv/CP ternary complexes they were 7.9–8.4 × 10^12^/L and 13.7–14.7 g/dL, respectively (lower values for the binary complex). These differences occurred in healthy rats due to some kidney problems that were more affected by the treatment with the CP solution (anemia), less affected after treatment with the γ-CD/CP binary complex, and almost not affected by using the antioxidant-protected γ-CD/Rv/CP ternary complex. Similar behavior was observed by using the β-CD/CP binary complex and β-CD/*Ficaria verna* Huds. extract/CP ternary complex for the treatment of healthy Wistar rats [47]. However, the differences were minor. The HCT parameter also had lower values after the γ-CD/CP binary complex treatment, which were at the limit of the normal values in healthy rats (35–52%) [43,44,45,46]. The difference was in accordance with the correlation of HCT with the RBC and HGB parameters. PLT and PCT had slightly lower values for the γ-CD/CP-treated group; however, they were in the normal range (500–1370 × 10^9^/L for PLT). These decreases could be due to the medication conditions [44].

Regarding the liver-related biochemical parameters, all values for the non-antioxidant-protected group (treated with the γ-CD/CP binary complex, without the protection of Rv) were significantly higher than the control group. On the other hand, the antioxidant-protected group that was treated with the γ-CD/Rv/CP ternary complex had liver-related biochemical parameter values closer to the control group or even lower. The GOT/AST parameter value was 107 IU/L in the CP-treated group, much higher than the normal limit in healthy rats (32.8–53 IU/L) [45,48]. On the contrary, the control and γ-CD/Rv/CP ternary complex-treated groups had GOT/AST values of only 73.8 and 65.8 IU/L, respectively, which were in the normal range. A similar situation was observed for the GGT parameter, with a value for the γ-CD/CP-treated group of 15.17 IU/L, in comparison with the control and γ-CD/Rv/CP-treated groups that had lower values, especially for the case of the ternary complex (9.83 IU/L, which was in the normal range of <20 IU/L). For the GPT/ALT and T-Bil (total bilirubin) parameters, the highest values were obtained for the same non-antioxidant-protected γ-CD/CP-treated group (68.17 IU/L and 0.70 mg/dL, respectively), while the antioxidant-protected γ-CD/Rv/CP ternary complex cases revealed intermediate values by comparison with the control group. High levels of GOT/AST, GPT/ALT, and GGT are signs of liver damage, which are more obvious for the treatment with the γ-CD/CP binary complex, which has no antioxidant protection effect. The co-encapsulation of antioxidant Rv into the ternary complex significantly reduced the toxic effect of the CP. It is well known that CP-induced toxicities are mainly due to the formation of free radicals. They further lead to the oxidative damage of various organs, especially the liver and kidney [13]. The “on-site” presence of the antioxidant Rv with radical scavenging activity (co-encapsulated with CP) partially protects these organs against CP-related damage. High total bilirubin (T-Bil) revealed the temporary stress of the liver due to the administration of CP. The presence of Rv in the ternary complex significantly reduced the level of liver stress [48]. However, the Tukey HSD test values for the liver-related biochemical parameters revealed no significant differences between the groups in the present study based on a single injection technique. Further studies on sub-chronic treatments can be more conclusive in this regard.

Among the kidney-related biochemical parameters, BUN, UA, and Cre were the most affected after treatment with the γ-CD/CP binary complex. However, the corresponding parameters for the group treated with the Rv-protected γ-CD/Rv/CP ternary complex had intermediate values that were closer to the control group. For the γ-CD/CP binary complex group, these values were 16.67, 2.65, and 0.80 mg/dL, while for the γ-CD/Rv/CP ternary complex group, the values were lower (14.17, 1.20, and 0.60 mg/dL), closer to the values corresponding to the control group (12.50, 1.10, and 0.55 mg/dL). These values demonstrate the harmful effect of the non-antioxidant-protected binary complex treatment, by affecting the normal function of the kidneys (UA value exceeding the normal range of 0.80–1.90 mg/dL) or even by blocking this organ (high value for creatinine). On the other hand, the protein-related parameters, as well as Ca, Mg, and IP, were not significantly affected; however, the values for the γ-CD/CP binary complex-treated group were the highest. These findings demonstrate the beneficial effect of the presence of the antioxidant Rv in the CP-based solution used for the treatment of cancer. The host–guest co-encapsulation of Rv and CP in γ-CD was energetically favorable, as was proved by the theoretical molecular modeling and docking experiments.

**Figure 5:**
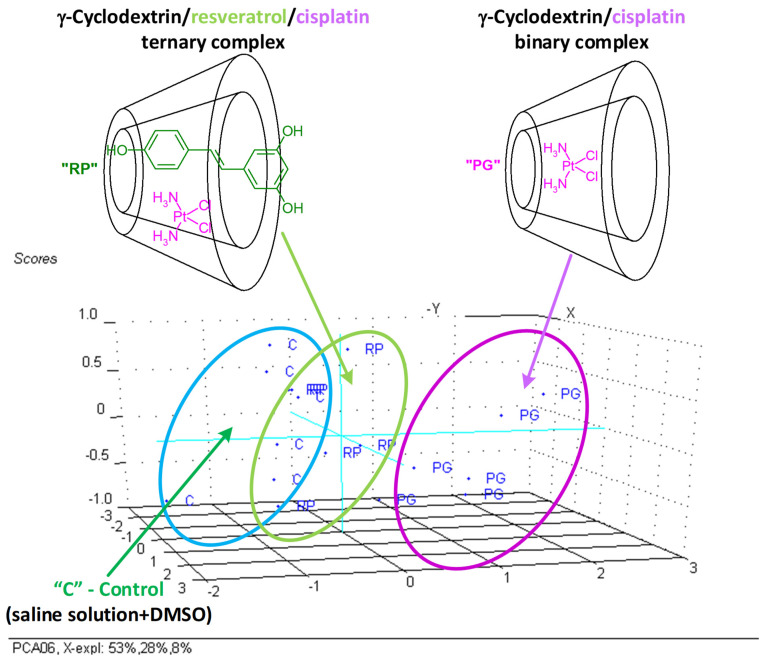
PC_2_, PC_3_ versus PC_1_ scores plot (3D representation) from the PCA analysis of the significant biochemical parameters (T-Bil, UA, Cre, T-Pro, Alb, Ca, Mg, and IP) determined from the Wistar rat plasma samples (“C”—control group, “PG”—group treated with γ-cyclodextrin/cisplatin binary complex, “RP”—group treated with γ-cyclodextrin/resveratrol/cisplatin ternary complex).

The PCA analysis allow identifying the most important parameters that discriminate the non-antioxidant-protected γ-CD/CP binary complex group from the Rv antioxidant-protected (treated with the γ-CD/Rv/CP ternary complex) and control groups [34,49,50,51]. The first group was clearly classified and discriminated, especially by the IP and UA parameters, again demonstrating the importance of antioxidant protection against liver and kidney damage determined by CP administration. Additionally, γ-CD can also protect these organs by a controlled release of bioactive compounds, as was observed by the partial superposition of the γ-CD/Rv/CP ternary complex and control classes by PCA (Figure 5).

## 5. Conclusions

The overall conclusion of this first study on the effects of resveratrol and γ-cyclodextrin on the hematological and biochemical parameters of healthy Wistar rats treated with cisplatin is that resveratrol plays an important role in protecting the organs, especially the kidneys. The Wistar rats treated with the γ-cyclodextrin/resveratrol/cisplatin ternary complex in solution had biochemical parameter values close to normal ranges, in comparison with the rats only treated with the γ-cyclodextrin/cisplatin binary complex, where the values were significantly higher for uric acid and creatinine. The post hoc Tukey HSD test (a pairwise comparison technique) was the most appropriate EDA statistical technique for the evaluation of the significant statistical differences between the antioxidant-protected and non-antioxidant-protected groups, producing very good results for the abovementioned kidney-related parameters (*p* < 0.005). These findings help to develop cisplatin-based cancer treatment protocols with significantly reduced harmful effects. Moreover, this study presents new idea on the safely treatment of various cancer types using (organo)metallic antineoplastic agents.

## Figures and Tables

**Table 1:** Wistar rat groups (six groups of *n* = 6 animals) used for the experimental design (see Section 2.3 for codes). Animal weight and the volume of the injected solution are expressed as mean ± standard deviation, SD (*n* = 6).

Code	Male/Female Ratio	Weight (g)	Solution Volume (mL)
“C”	4/2	254.8 ± 23.9	2.55 ± 0.24
“P”	2/4	266.8 ± 37.3	2.67 ± 0.37
“G”	2/4	240.3 ± 31.9	2.40 ± 0.32
“PG”	2/4	246.3 ± 61.3	2.46 ± 0.61
“RG”	2/4	254.0 ± 23.0	2.54 ± 0.23
“RP”	2/4	235.3 ± 45.1	2.35 ± 0.45

**Table 2:** Interaction energies (kcal/mol) of γ-CD/CP, γ-CD/Rv binary complexes (codes “PG” and “RG”) and γ-CD/Rv/CP ternary complex (code “RP”), determined by MM+ geometry optimization in a vacuum. Energy of the “empty” γ-CD molecule at the most stable conformation is 91.29 kcal/mol.

Code	Energy of the Guest Molecules (kcal/mol)	Energy of the Complex (kcal/mol)	Host–Guest Interaction Energy (kcal/mol)
“PG”	1.84	82.63	10.50
“RG”	−4.44	68.92	17.93
“RP”	−2.6	62.21	26.48

## Data Availability

Data are contained within the article and Supplementary Materials.

## Supplementary Materials

The following supporting information can be downloaded at https://www.mdpi.com/article/10.3390/biomedicines11102726/s1, Table S1: results for the hematological parameters determined from the Wistar rat blood samples (“C”—control group, “P”—group treated with cisplatin, “G”—group treated with “empty” γ-cyclodextrin, “PG”—group treated with γ-cyclodextrin/cisplatin binary complex, “RG”—group treated with γ-cyclodextrin/resveratrol binary complex, “RP”—group treated with γ-cyclodextrin/resveratrol/cisplatin ternary complex); values are presented as means ± standard deviations, SD for *n* = 6; Table S2: results for the liver-related biochemical parameters determined from the Wistar rat plasma samples (“C”—control group, “PG”—group treated with γ-cyclodextrin/cisplatin binary complex, “RP”—group treated with γ-cyclodextrin/resveratrol/cisplatin ternary complex); values are presented as means ± standard deviations, SD for *n* = 6; Table S3: results for the kidney-related biochemical parameters determined from the Wistar rat plasma samples (“C”—control group, “PG”—group treated with γ-cyclodextrin/cisplatin binary complex, “RP”—group treated with γ-cyclodextrin/resveratrol/cisplatin ternary complex); values are presented as means ± standard deviations, SD for *n* = 6; Table S4: results for the other biochemical parameters determined from the Wistar rat plasma samples (“C”—control group, “PG”—group treated with γ-cyclodextrin/cisplatin binary complex, “RP”—group treated with γ-cyclodextrin/resveratrol/cisplatin ternary complex); values are presented as means ± standard deviations, SD for *n* = 6; Table S5: Significant *p*-levels from the Tukey HSD test for WBC (white blood cell, 10^9^/L) parameter values determined from the Wistar rat blood samples (“C”—control group, “P”—group treated with cisplatin, “G”—group treated with “empty” γ-cyclodextrin, “PG”—group treated with γ-cyclodextrin/cisplatin binary complex, “RG”—group treated with γ-cyclodextrin/resveratrol binary complex, “RP”—group treated with γ-cyclodextrin/resveratrol/cisplatin ternary complex; *n* = 6); Table S6: Significant *p*-levels from the Tukey HSD test for LYM (lymphocyte, 10^9^/L) parameter values determined from the Wistar rat blood samples (“C”—control group, “P”—group treated with cisplatin, “G”—group treated with “empty” γ-cyclodextrin, “PG”—group treated with γ-cyclodextrin/cisplatin binary complex, “RG”—group treated with γ-cyclodextrin/resveratrol binary complex, “RP”—group treated with γ-cyclodextrin/resveratrol/cisplatin ternary complex; *n* = 6). *p*-level values lower than 0.05 are in bold; Table S7: Significant *p*-levels from the Tukey HSD test for MID (middle cell, 10^9^/L) parameter values determined from the Wistar rat blood samples (“C”—control group, “P”—group treated with cisplatin, “G”—group treated with “empty” γ-cyclodextrin, “PG”—group treated with γ-cyclodextrin/cisplatin binary complex, “RG”—group treated with γ-cyclodextrin/resveratrol binary complex, “RP”—group treated with γ-cyclodextrin/resveratrol/cisplatin ternary complex; *n* = 6). *p*-level values lower than 0.05 are in bold; Table S8: Significant *p*-levels from the Tukey HSD test for GRA (granulocyte, 10^9^/L) parameter values determined from the Wistar rat blood samples (“C”—control group, “P”—group treated with cisplatin, “G”—group treated with “empty” γ-cyclodextrin, “PG”—group treated with γ-cyclodextrin/cisplatin binary complex, “RG”—group treated with γ-cyclodextrin/resveratrol binary complex, “RP”—group treated with γ-cyclodextrin/resveratrol/cisplatin ternary complex; *n* = 6); Table S9: Significant *p*-levels from the Tukey HSD test for LY% (lymphocyte, %) parameter values determined from the Wistar rat blood samples (“C”—control group, “P”—group treated with cisplatin, “G”—group treated with “empty” γ-cyclodextrin, “PG”—group treated with γ-cyclodextrin/cisplatin binary complex, “RG”—group treated with γ-cyclodextrin/resveratrol binary complex, “RP”—group treated with γ-cyclodextrin/resveratrol/cisplatin ternary complex; *n* = 6); Table S10: Significant *p*-levels from the Tukey HSD test for MI% (middle cell, %) parameter values determined from the Wistar rat blood samples (“C”—control group, “P”—group treated with cisplatin, “G”—group treated with “empty” γ-cyclodextrin, “PG”—group treated with γ-cyclodextrin/cisplatin binary complex, “RG”—group treated with γ-cyclodextrin/resveratrol binary complex, “RP”—group treated with γ-cyclodextrin/resveratrol/cisplatin ternary complex; *n* = 6); Table S11: Significant *p*-levels from the Tukey HSD test for GR% (granulocyte, %) parameter values determined from the Wistar rat blood samples (“C”—control group, “P”—group treated with cisplatin, “G”—group treated with “empty” γ-cyclodextrin, “PG”—group treated with γ-cyclodextrin/cisplatin binary complex, “RG”—group treated with γ-cyclodextrin/resveratrol binary complex, “RP”—group treated with γ-cyclodextrin/resveratrol/cisplatin ternary complex; *n* = 6); Table S12: Significant *p*-levels from the Tukey HSD test for RBC (red blood cell, 10^12^/L) parameter values determined from the Wistar rat blood samples (“C”—control group, “P”—group treated with cisplatin, “G”—group treated with “empty” γ-cyclodextrin, “PG”—group treated with γ-cyclodextrin/cisplatin binary complex, “RG”—group treated with γ-cyclodextrin/resveratrol binary complex, “RP”—group treated with γ-cyclodextrin/resveratrol/cisplatin ternary complex; *n* = 6); Table S13: Significant *p*-levels from the Tukey HSD test for HGB (hemoglobin, g/dL) parameter values determined from the Wistar rat blood samples (“C”—control group, “P”—group treated with cisplatin, “G”—group treated with “empty” γ-cyclodextrin, “PG”—group treated with γ-cyclodextrin/cisplatin binary complex, “RG”—group treated with γ-cyclodextrin/resveratrol binary complex, “RP”—group treated with γ-cyclodextrin/resveratrol/cisplatin ternary complex; *n* = 6); Table S14: Significant *p*-levels from the Tukey HSD test for HCT (hematocrit, %) parameter values determined from the Wistar rat blood samples (“C”—control group, “P”—group treated with cisplatin, “G”—group treated with “empty” γ-cyclodextrin, “PG”—group treated with γ-cyclodextrin/cisplatin binary complex, “RG”—group treated with γ-cyclodextrin/resveratrol binary complex, “RP”—group treated with γ-cyclodextrin/resveratrol/cisplatin ternary complex; *n* = 6); Table S15: Significant *p*-levels from the Tukey HSD test for MCV (mean corpuscular volume, fL) parameter values determined from the Wistar rat blood samples (“C”—control group, “P”—group treated with cisplatin, “G”—group treated with “empty” γ-cyclodextrin, “PG”—group treated with γ-cyclodextrin/cisplatin binary complex, “RG”—group treated with γ-cyclodextrin/resveratrol binary complex, “RP”—group treated with γ-cyclodextrin/resveratrol/cisplatin ternary complex; *n* = 6); Table S16: Significant *p*-levels from the Tukey HSD test for MCH (mean corpuscular hemoglobin, pg) parameter values determined from the Wistar rat blood samples (“C”—control group, “P”—group treated with cisplatin, “G”—group treated with “empty” γ-cyclodextrin, “PG”—group treated with γ-cyclodextrin/cisplatin binary complex, “RG”—group treated with γ-cyclodextrin/resveratrol binary complex, “RP”—group treated with γ-cyclodextrin/resveratrol/cisplatin ternary complex; *n* = 6); Table S17: Significant *p*-levels from the Tukey HSD test for MCHC (mean corpuscular hemoglobin concentration, g/dL) parameter values determined from the Wistar rat blood samples (“C”—control group, “P”—group treated with cisplatin, “G”—group treated with “empty” γ-cyclodextrin, “PG”—group treated with γ-cyclodextrin/cisplatin binary complex, “RG”—group treated with γ-cyclodextrin/resveratrol binary complex, “RP”—group treated with γ-cyclodextrin/resveratrol/cisplatin ternary complex; *n* = 6); Table S18: Significant *p*-levels from the Tukey HSD test for RDWc (red blood cell distribution width, %) parameter values determined from the Wistar rat blood samples (“C”—control group, “P”—group treated with cisplatin, “G”—group treated with “empty” γ-cyclodextrin, “PG”—group treated with γ-cyclodextrin/cisplatin binary complex, “RG”—group treated with γ-cyclodextrin/resveratrol binary complex, “RP”—group treated with γ-cyclodextrin/resveratrol/cisplatin ternary complex; *n* = 6); Table S19: Significant *p*-levels from the Tukey HSD test for PLT (platelet/thrombocyte, 10^9^/L) parameter values determined from the Wistar rat blood samples (“C”—control group, “P”—group treated with cisplatin, “G”—group treated with “empty” γ-cyclodextrin, “PG”—group treated with γ-cyclodextrin/cisplatin binary complex, “RG”—group treated with γ-cyclodextrin/resveratrol binary complex, “RP”—group treated with γ-cyclodextrin/resveratrol/cisplatin ternary complex; *n* = 6); Table S20: Significant *p*-levels from the Tukey HSD test for PCT (plateletcrit, %) parameter values determined from the Wistar rat blood samples (“C”—control group, “P”—group treated with cisplatin, “G”—group treated with “empty” γ-cyclodextrin, “PG”—group treated with γ-cyclodextrin/cisplatin binary complex, “RG”—group treated with γ-cyclodextrin/resveratrol binary complex, “RP”—group treated with γ-cyclodextrin/resveratrol/cisplatin ternary complex; *n* = 6); Table S21: Significant *p*-levels from the Tukey HSD test for MPV (mean platelet volume, fL) parameter values determined from the Wistar rat blood samples (“C”—control group, “P”—group treated with cisplatin, “G”—group treated with “empty” γ-cyclodextrin, “PG”—group treated with γ-cyclodextrin/cisplatin binary complex, “RG”—group treated with γ-cyclodextrin/resveratrol binary complex, “RP”—group treated with γ-cyclodextrin/resveratrol/cisplatin ternary complex; *n* = 6); Table S22: Significant *p*-levels from the Tukey HSD test for PDWc (platelet distribution Width, %) parameter values determined from the Wistar rat blood samples (“C”—control group, “P”—group treated with cisplatin, “G”—group treated with “empty” γ-cyclodextrin, “PG”—group treated with γ-cyclodextrin/cisplatin binary complex, “RG”—group treated with γ-cyclodextrin/resveratrol binary complex, “RP”—group treated with γ-cyclodextrin/resveratrol/cisplatin ternary complex; *n* = 6); Table S23: Significant *p*-levels from the Tukey HSD test for GOT/AST (glutamic-oxalacetic transaminase/aspartate transaminase, expressed as IU/L) parameter values determined from the Wistar rat plasma samples (“C”—control group, “PG”—group treated with γ-cyclodextrin/cisplatin binary complex, “RP”—group treated with γ-cyclodextrin/resveratrol/cisplatin ternary complex; *n* = 6); Table S24: Significant *p*-levels from the Tukey HSD test for GPT/ALT (glutamic-pyruvic transaminase/alanine transaminase, IU/L) parameter values determined from the Wistar rat plasma samples (“C”—control group, “PG”—group treated with γ-cyclodextrin/cisplatin binary complex, “RP”—group treated with γ-cyclodextrin/resveratrol/cisplatin ternary complex; *n* = 6); Table S25: Significant *p*-levels from the Tukey HSD test for GGT (gamma-glutamyl transferase, IU/L) parameter values determined from the Wistar rat plasma samples (“C”—control group, “PG”—group treated with γ-cyclodextrin/cisplatin binary complex, “RP”—group treated with γ-cyclodextrin/resveratrol/cisplatin ternary complex; *n* = 6); Table S26: Significant *p*-levels from the Tukey HSD test for T-Bil (total bilirubin, mg/dL) parameter values determined from the Wistar rat plasma samples (“C”—control group, “PG”—group treated with γ-cyclodextrin/cisplatin binary complex, “RP”—group treated with γ-cyclodextrin/resveratrol/cisplatin ternary complex; *n* = 6); Table S27: Significant *p*-levels from the Tukey HSD test for BUN (blood urea nitrogen, mg/dL) parameter values determined from the Wistar rat plasma samples (“C”—control group, “PG”—group treated with γ-cyclodextrin/cisplatin binary complex, “RP”—group treated with γ-cyclodextrin/resveratrol/cisplatin ternary complex; *n* = 6). *p*-level values lower than 0.05 are in bold; Table S28: Significant *p*-levels from the Tukey HSD test for UA (uric acid, mg/dL) parameter values determined from the Wistar rat plasma samples (“C”—control group, “PG”—group treated with γ-cyclodextrin/cisplatin binary complex, “RP”—group treated with γ-cyclodextrin/resveratrol/cisplatin ternary complex; *n* = 6). *p*-level values lower than 0.05 are in bold; Table S29: Significant *p*-levels from the Tukey HSD test for Cre (creatinine, mg/dL) parameter values determined from the Wistar rat plasma samples (“C”—control group, “PG”—group treated with γ-cyclodextrin/cisplatin binary complex, “RP”—group treated with γ-cyclodextrin/resveratrol/cisplatin ternary complex; *n* = 6). *p*-level values lower than 0.05 are in bold; Table S30: Significant *p*-levels from the Tukey HSD test for T-Pro (total protein, g/dL) parameter values determined from the Wistar rat plasma samples (“C”—control group, “PG”—group treated with γ-cyclodextrin/cisplatin binary complex, “RP”—group treated with γ-cyclodextrin/resveratrol/cisplatin ternary complex; *n* = 6). *p*-level values lower than 0.05 are in bold; Table S31: Significant *p*-levels from the Tukey HSD test for Alb (albumin, g/dL) parameter values determined from the Wistar rat plasma samples (“C”—control group, “PG”—group treated with γ-cyclodextrin/cisplatin binary complex, “RP”—group treated with γ-cyclodextrin/resveratrol/cisplatin ternary complex; *n* = 6). *p*-level values lower than 0.05 are in bold; Table S32: Significant *p*-levels from the Tukey HSD test for ALP (alkaline phosphatase, IU/L) parameter values determined from the Wistar rat plasma samples (“C”—control group, “PG”—group treated with γ-cyclodextrin/cisplatin binary complex, “RP”—group treated with γ-cyclodextrin/resveratrol/cisplatin ternary complex; *n* = 6); Table S33: Significant *p*-levels from the Tukey HSD test for CPK (creatine phosphokinase, IU/L) parameter values determined from the Wistar rat plasma samples (“C”—control group, “PG”—group treated with γ-cyclodextrin/cisplatin binary complex, “RP”—group treated with γ-cyclodextrin/resveratrol/cisplatin ternary complex; *n* = 6). *p*-level values lower than 0.05 are in bold; Table S34: Significant *p*-levels from the Tukey HSD test for calcium (Ca, mg/dL) parameter values determined from the Wistar rat plasma samples (“C”—control group, “PG”—group treated with γ-cyclodextrin/cisplatin binary complex, “RP”—group treated with γ-cyclodextrin/resveratrol/cisplatin ternary complex; *n* = 6); Table S35: Significant *p*-levels from the Tukey HSD test for magnesium (Mg, mg/dL) parameter values determined from the Wistar rat plasma samples (“C”—control group, “PG”—group treated with γ-cyclodextrin/cisplatin binary complex, “RP”—group treated with γ-cyclodextrin/resveratrol/cisplatin ternary complex; *n* = 6). *p*-level values lower than 0.05 are in bold; Table S36: Significant *p*-levels from the Tukey HSD test for inorganic phosphorus (IP, mg/dL) parameter values determined from the Wistar rat plasma samples (“C”—control group, “PG”—group treated with γ-cyclodextrin/cisplatin binary complex, “RP”—group treated with γ-cyclodextrin/resveratrol/cisplatin ternary complex; *n* = 6). *p*-level values lower than 0.05 are in bold; Figure S1: PC_2_ versus PC_1_ scores plot from the PCA analysis of the hematological parameters determined from the Wistar rat blood samples (“C”—control group, “P”—group treated with cisplatin, “G”—group treated with “empty” γ-cyclodextrin, “PG”—group treated with γ-cyclodextrin/cisplatin binary complex, “RG”—group treated with γ-cyclodextrin/resveratrol binary complex, “RP”—group treated with γ-cyclodextrin/resveratrol/cisplatin ternary complex); Figure S2: PC_2_ versus PC_1_ loadings plot from the PCA analysis of the hematological parameters determined from the Wistar rat blood samples; Figure S3: PC_2_ versus PC_1_ scores plot from the PCA analysis of the hematological parameters (excepting PLT, GR% and LY%) determined from the Wistar rat blood samples (“C”—control group, “P”—group treated with cisplatin, “G”—group treated with “empty” γ-cyclodextrin, “PG”—group treated with γ-cyclodextrin/cisplatin binary complex, “RG”—group treated with γ-cyclodextrin/resveratrol binary complex, “RP”—group treated with γ-cyclodextrin/resveratrol/cisplatin ternary complex); Figure S4: PC_2_ versus PC_1_ loadings plot from the PCA analysis of the hematological parameters (except PLT, GR%, and LY%) determined from the Wistar rat blood samples; Figure S5: PC_2_ versus PC_1_ scores plot from the PCA analysis of the hematological parameters (only LYM, MID, GRA, RBC, HGB, HCT, MCH, MCHC, RDWc, PCT, MPV, and PDWc) determined from the Wistar rat blood samples (only “C”—control group, “PG”—group treated with γ-cyclodextrin/cisplatin binary complex, and “RP”—group treated with γ-cyclodextrin/resveratrol/cisplatin ternary complex); Figure S6: PC_2_ versus PC_1_ loadings plot from the PCA analysis of the hematological parameters (only LYM, MID, GRA, RBC, HGB, HCT, MCH, MCHC, RDWc, PCT, MPV, and PDWc) determined from the Wistar rat blood samples (only “C”—control group, “PG”—group treated with γ-cyclodextrin/cisplatin binary complex, and “RP”—group treated with γ-cyclodextrin/resveratrol/cisplatin ternary complex); Figure S7: PC_2_ versus PC_1_ scores plot from the PCA analyses of all biochemical parameters determined from the Wistar rat plasma samples (“C”—control group, “PG”—group treated with γ-cyclodextrin/cisplatin binary complex, “RP”—group treated with γ-cyclodextrin/resveratrol/cisplatin ternary complex); Figure S8: PC_2_ versus PC_1_ loadings plot from the PCA analyses of all biochemical parameters determined from the Wistar rat plasma samples; Figure S9: PC_2_ versus PC_1_ scores plot from the PCA analysis of biochemical parameters (excepting ALP and CPK) determined from the Wistar rat plasma samples (“C”—control group, “PG”—group treated with γ-cyclodextrin/cisplatin binary complex, “RP”—group treated with γ-cyclodextrin/resveratrol/cisplatin ternary complex); Figure S10: PC_2_ versus PC_1_ loadings plot from the PCA analysis of biochemical parameters (except ALP and CPK) determined from the Wistar rat plasma samples; Figure S11: PC_2_ versus PC_1_ scores plot from the PCA analysis of biochemical parameters (except ALP, CPK, GOT/AST, and GPT/ALT) determined from the Wistar rat plasma samples (“C”—control group, “PG”—group treated with γ-cyclodextrin/cisplatin binary complex, “RP”—group treated with γ-cyclodextrin/resveratrol/cisplatin ternary complex); Figure S12: PC_2_ versus PC_1_ loadings plot from the PCA analysis of biochemical parameters (except ALP, CPK, GOT/AST, and GPT/ALT) determined from the Wistar rat plasma samples; Figure S13: PC_2_ versus PC_1_ scores plot from the PCA analysis of all hematological and biochemical parameters determined from the Wistar rat blood or plasma samples (“C”—control group, “P”—group treated with cisplatin, “G”—group treated with “empty” γ-cyclodextrin, “PG”—group treated with γ-cyclodextrin/cisplatin binary complex, “RG”—group treated with γ-cyclodextrin/resveratrol binary complex, “RP”—group treated with γ-cyclodextrin/resveratrol/cisplatin ternary complex); Figure S14: PC_2_ versus PC_1_ loadings plot from the PCA analyses of all hematological and biochemical parameters determined from the Wistar rat blood or plasma samples (“C”—control group, “P”—group treated with cisplatin, “G”—group treated with “empty” γ-cyclodextrin, “PG”—group treated with γ-cyclodextrin/cisplatin binary complex, “RG”—group treated with γ-cyclodextrin/resveratrol binary complex, “RP”—group treated with γ-cyclodextrin/resveratrol/cisplatin ternary complex); Figure S15: PC_2_ versus PC_1_ scores plot from the PCA analysis of the significant biochemical parameters (T-Bil, UA, Cre, T-Pro, Alb, Ca, Mg, and IP) determined from the Wistar rat plasma samples (“C”—control group, “PG”—group treated with γ-cyclodextrin/cisplatin binary complex, “RP”—group treated with γ-cyclodextrin/resveratrol/cisplatin ternary complex); Figure S16: PC_2_ versus PC_1_ loadings plot from the PCA analysis of the significant biochemical parameters (T-Bil, UA, Cre, T-Pro, Alb, Ca, Mg, and IP) determined from the Wistar rat plasma samples; Figure S17: molecular-modeled γ-cyclodextrin/resveratrol binary complex at the minimum energy stage, obtained by MM+ geometry optimization; guest compound is in bold; Figure S18: molecular-modeled γ-cyclodextrin/cisplatin binary complex at the minimum energy stage, obtained by MM+ geometry optimization; guest compound is in bold.
