# Supplementary material for: Beneficial Effects of Resveratrol and γ-Cyclodextrin on the Hematological and Biochemical Parameters of Healthy Wistar Rats Treated with Cisplatin: A PCA Approach
Source: Biomedicines. 2023 Oct 8;11(10):2726. doi: 10.3390/biomedicines11102726 (PMC10604837; doi:10.3390/biomedicines11102726)
Supplement: Supplementary file 1 [file biomedicines-11-02726-s001.zip › biomedicines-2573264-supplementary.pdf]

*Article*

# Beneficial Effects of Resveratrol and γ-Cyclodextrin on the Hematological and Biochemical Parameters of Healthy Wistar Rats Treated with Cisplatin: A PCA Approach *Supplementary Material*

**Nicoleta-Gabriela Hădărugă [1,*], Zeno Gârban [2], Cornel Baltă [3], Florin Muselin [4], Daniel-Ioan Hădărugă [5] and Mircea Riviş [6]**

[1] Department of Food Science, University of Life Sciences "King Mihai I" from Timişoara, Calea Aradului 119, 300645-Timişoara, Romania; nicoletahadaruga@usvt.ro; nico_hadaruga@yahoo.com (N.G.H.)
[2] Working Group for Xenobiochemistry, Romanian Academy – Timişoara Branch, Mihai Viteazu Bd. 24, 300223-Timişoara, Romania; zeno.garban@yahoo.com (Z.G.)
[3] "Aurel Ardelean" Institute of Life Sciences, "Vasile Goldiş" Western University, Liviu Rebreanu 86, 310414-Arad, Romania; balta.cornel@uvvg.ro (C.B.)
[4] Department of Toxicology and Toxicoses, Plant Biology and Medicinal Plants, University of Life Sciences "King Mihai I" from Timișoara, Calea Aradului 119, 300645-Timisoara, Romania; florinmuselin@usvt.ro (F.M.)
[5] Department of Applied Chemistry, Organic and Natural Compounds Engineering, Polytechnic University of Timişoara, Carol Telbisz 6, 300001-Timişoara, Romania; daniel.hadaruga@upt.ro (D.I.H.)
[6] Department of Anesthesiology and Oral Surgery, "Victor Babeş" University of Medicine and Pharmacy Timişoara, Eftimie Murgu Sq. 2, 300041-Timişoara, Romania; mrivis@yahoo.com (M.R.)
* Correspondence: nicoletahadaruga@usvt.ro; nico_hadaruga@yahoo.com; Tel.: +40-256-277-423

## 1. Hematological parameters

**Table S1.** Results for the hematological parameters determined from the Wistar rat blood samples ("C" – control group, "P" – group treated with cisplatin, "G" – group treated with "empty" γ-cyclodextrin, "PG" – group treated with γ-cyclodextrin/cisplatin binary complex, "RG" – group treated with γ-cyclodextrin/resveratrol binary complex, "RP" – group treated with γ-cyclodextrin/resveratrol/cisplatin ternary complex); values are presented as mean ± standard deviation, SD for $n = 6$.

| Code | WBC [1] | LYM [2] | MID [3] | GRA [4] | LY% [5] | MI% [6] |
|---|---|---|---|---|---|---|
| "C" | 11.18±5.71 | 6.97±3.03 | 0.41±0.28 | 3.80±2.78 | 64.15±6.88 | 4.32±2.45 |
| "P" | 11.29±5.79 | 8.46±4.49 | 0.48±0.22 | 2.36±1.42 | 75.12±6.23 | 4.90±1.78 |
| "G" | 11.17±1.88 | 7.54±1.74 | 0.44±0.26 | 3.18±0.93 | 67.35±7.49 | 4.32±2.79 |
| "PG" | 10.63±2.65 | 7.25±1.53 | 0.47±0.24 | 2.91±1.06 | 68.92±4.34 | 4.20±1.97 |
| "RG" | 8.12±2.22 | 5.70±2.04 | 0.09±0.05 | 2.34±0.49 | 68.98±6.71 | 1.10±0.52 |
| "RP" | 8.12±2.37 | 5.19±1.93 | 0.24±0.15 | 2.68±1.00 | 63.30±8.10 | 3.40±2.72 |
| Normal range [43-46] | 2.1-19.5 | 2.0-14.1 | 0.00-0.98 | 0.1-5.4 | 55.0-97.0 | 0.0-5.0 |

| Code | GR% [7] | RBC [8] | HGB [9] | HCT [10] | MCV [11] | MCH [12] |
|---|---|---|---|---|---|---|
| "C" | 31.53±8.15 | 8.51±0.72 | 14.17±1.03 | 40.88±2.13 | 48.00±1.79 | 16.68±0.45 |
| "P" | 19.97±6.26 | 7.10±2.67 | 12.30±5.13 | 35.95±14.07 | 50.33±2.73 | 16.65±2.22 |
| "G" | 28.35±6.58 | 8.69±0.17 | 14.83±0.48 | 42.66±1.05 | 49.17±2.04 | 17.07±0.71 |
| "PG" | 26.88±3.87 | 8.43±0.55 | 14.70±0.42 | 41.83±2.06 | 49.50±1.52 | 17.48±0.85 |
| "RG" | 29.93±6.49 | 8.41±0.46 | 14.28±0.41 | 41.34±2.09 | 49.17±1.17 | 17.00±0.66 |
| "RP" | 33.32±7.70 | 7.92±0.24 | 13.70±0.76 | 39.35±2.08 | 49.67±2.42 | 17.30±0.82 |
| Normal range [43-46] | 2.00-31.00 | 5.30-10.00 | 14.00-18.00 | 35.00-52.00 | 50.00-62.00 | 16.00-23.00 |

| Code | MCHC 13 | RDWc [14] | PLT [15] | PCT [16] | MPV [17] | PDWc [18] |
|---|---|---|---|---|---|---|
| "C" | 34.62±0.97 | 18.30±1.44 | 690.8±205.4 | 0.40±0.12 | 5.87±0.31 | 30.22±1.36 |
| "P" | 33.15±3.53 | 16.42±0.96 | 663.0±486.0 | 0.39±0.29 | 5.98±0.39 | 30.10±1.71 |
| "G" | 34.78±1.12 | 16.97±0.85 | 714.3±95.1 | 0.41±0.05 | 5.75±0.28 | 29.07±0.49 |
| "PG" | 35.15±1.35 | 16.93±0.88 | 695.8±88.3 | 0.40±0.05 | 5.70±0.21 | 29.23±0.98 |
| "RG" | 34.57±1.04 | 17.20±1.84 | 946.5±213.5 | 0.58±0.15 | 6.08±0.21 | 31.07±1.20 |
| "RP" | 34.78±0.51 | 17.15±1.67 | 798.8±75.3 | 0.47±0.05 | 5.87±0.18 | 29.80±0.73 |
| Normal range [43-46] | 31.00-40.00 | - | 500.0-1370.0 | - | 5.00-20.00 | - |

[1] WBC – White Blood Cells, expressed as $10^9$/L; [2] LYM – Lymphocytes, $10^9$/L; [3] MID – Middle Cells, $10^9$/L; [4] GRA – Granulocytes, $10^9$/L; [5] LY% – Lymphocytes, %; [6] MI% – Middle Cells, %; [7] GR% – Granulocytes, %; [8] RBC – Red Blood Cells, $10^{12}$/L; [9] HGB – Hemoglobin, g/dL; [10] HCT – Hematocrit, %; [11] MCV – Mean Corpuscular Volume, fL; [12] MCH – Mean Corpuscular Hemoglobin, pg; [13] MCHC – Mean Corpuscular Hemoglobin Concentration, g/dL; [14] RDWc – Red Blood Cell Distribution Width, %; [15] PLT – Platelet/Thrombocytes, $10^9$/L; [16] PCT – Plateletcrit, %; [17] MPV – Mean Platelet Volume, fL; [18] PDWc – Platelet Distribution Width, %.

## 2. Biochemical parameters

**Table S2.** Results for the liver-related biochemical parameters determined from the Wistar rat plasma samples ("C" – control group, "PG" – group treated with γ-cyclodextrin/cisplatin binary complex, "RP" – group treated with γ-cyclodextrin/resveratrol/cisplatin ternary complex); values are presented as mean ± standard deviation, SD for *n* = 6.

| Code | GOT/AST [1] | GPT/ALT [2] | GGT [3] | T-Bil [4] |
|---|---|---|---|---|
| "C" | 73.83±52.58 | 50.50±22.28 | 12.83±4.71 | 0.33±0.28 |
| "PG" | 107.33±39.74 | 68.17±23.74 | 15.17±8.26 | 0.70±0.55 |
| "RP" | 65.83±13.82 | 56.50±15.12 | 9.83±0.41 | 0.40±0.06 |
| Normal range [43-46] | 32.80-80.80 | 17.50-80.00 | <20.00 | 0.20-0.55 |

[1] GOT/AST – glutamic-oxalacetic transaminase / aspartate transaminase, expressed as IU/L;
[2] GPT/ALT – glutamic-pyruvic transaminase / alanine transaminase, IU/L;
[3] GGT – gamma-glutamyl transferase, IU/L;
[4] T-Bil – Total Bilirubin, mg/dL.

**Table S3.** Results for the kidney-related biochemical parameters determined from the Wistar rat plasma samples ("C" – control group, "PG" – group treated with γ-cyclodextrin/cisplatin binary complex, "RP" – group treated with γ-cyclodextrin/resveratrol/cisplatin ternary complex); values are presented as mean ± standard deviation, SD for *n* = 6.

| Code | BUN [1] | UA [2] | Cre [3] | T-Pro [4] | Alb [5] | ALP [6] | CPK [7] |
|---|---|---|---|---|---|---|---|
| "C" | 12.50±1.87 | 1.10±0.33 | 0.55±0.10 | 6.03±0.52 | 3.18±0.33 | 296.7±129.6 | 810.3* |
| "PG" | 16.67±2.88 | 2.65±0.08 | 0.80±0.07 | 7.20±0.50 | 3.73±0.23 | 439.0±240.4 | 603.3±222.8 |
| "RP" | 14.17±1.47 | 1.20±0.40 | 0.60±0.09 | 6.65±0.60 | 3.62±0.18 | 369.5±148.2 | 285.0±188.8 |
| Normal range [43-46] | 10.00-21.00 | 0.80-1.90 | 0.20-0.80 | 5.60-7.60 | 3.80-4.80 | 174.0-589.0 | - |

[1] BUN – Blood Urea Nitrogen, expressed as mg/dL;
[2] UA – Uric Acid, mg/dL;
[3] Cre – Creatinine, mg/dL;
[4] T-Pro – Total Proteins, g/dL;
[5] Alb – Albumins, g/dL;
[6] ALP – Alkaline Phosphatase, IU/L;
[7] CPK – Creatine Phosphokinase, IU/L.

**Table S4.** Results for the other biochemical parameters determined from the Wistar rat plasma samples ("C" – control group, "PG" – group treated with γ-cyclodextrin/cisplatin binary complex, "RP" – group treated with γ-cyclodextrin/resveratrol/cisplatin ternary complex); values are presented as mean ± standard deviation, SD for *n* = 6.

| Code | Ca [1] | Mg [2] | IP [3] |
|---|---|---|---|
| "C" | 11.85±0.54 | 2.12±0.15 | 4.48±0.59 |
| "PG" | 12.45±0.61 | 2.53±0.20 | 5.13±1.47 |
| "RP" | 11.85±0.37 | 2.05±0.14 | 3.02±1.30 |
| Normal range [43-46] | 5.30-13.00 | 2.70-3.50 | 3.11-11.00 |

[1] Ca – Calcium, mg/dL;
[2] Mg – Magnesium, mg/dL;
[3] IP – Inorganic phosphorus, mg/dL.

## 3. Tukey HSD tests for the hematological parameters

**Table S5.** Significant *p*-levels from the Tukey HSD test for the WBC (White Blood Cells, $10^9$/L) parameter values determined from the Wistar rat blood samples ("C" – control group, "P" – group treated with cisplatin, "G" – group treated with "empty" γ-cyclodextrin, "PG" – group treated with γ-cyclodextrin/cisplatin binary complex, "RG" – group treated with γ-cyclodextrin/resveratrol binary complex, "RP" – group treated with γ-cyclodextrin/resveratrol/cisplatin ternary complex; *n* = 6).

| | *"C"* | *"P"* | *"G"* | *"PG"* | *"RG"* | *"RP"* |
|---|---|---|---|---|---|---|
| *"C"* | | 0.906882 | 1.000000 | 0.999710 | 0.609120 | 0.606956 |
| *"P"* | | | 0.903990 | 0.783313 | 0.145368 | 0.144442 |
| *"G"* | | | | 0.999750 | 0.614526 | 0.612364 |
| *"PG"* | | | | | 0.781050 | 0.779184 |
| *"RG"* | | | | | | 1.000000 |
| *"RP"* | | | | | | |

**Table S6.** Significant *p*-levels from the Tukey HSD test for the LYM (Lymphocytes, $10^9$/L) parameter values determined from the Wistar rat blood samples ("C" – control group, "P" – group treated with cisplatin, "G" – group treated with "empty" γ-cyclodextrin, "PG" – group treated with γ-cyclodextrin/cisplatin binary complex, "RG" – group treated with γ-cyclodextrin/resveratrol binary complex, "RP" – group treated with γ-cyclodextrin/resveratrol/cisplatin ternary complex; *n* = 6). *p*-level values lower than 0.05 are bolded.

| | *"C"* | *"P"* | *"G"* | *"PG"* | *"RG"* | *"RP"* |
|---|---|---|---|---|---|---|
| *"C"* | | 0.297603 | 0.998000 | 0.999940 | 0.922307 | 0.749016 |
| *"P"* | | | 0.526067 | 0.402307 | **0.048163** | **0.020347** |
| *"G"* | | | | 0.999928 | 0.724219 | 0.487064 |
| *"PG"* | | | | | 0.840007 | 0.622545 |
| *"RG"* | | | | | | 0.998801 |
| *"RP"* | | | | | | |

**Table S7.** Significant *p*-levels from the Tukey HSD test for the MID (Middle Cells, $10^9$/L) parameter values determined from the Wistar rat blood samples ("C" – control group, "P" – group treated with cisplatin, "G" – group treated with "empty" γ-cyclodextrin, "PG" – group treated with γ-cyclodextrin/cisplatin binary complex, "RG" – group treated with γ-cyclodextrin/resveratrol binary complex, "RP" – group treated with γ-cyclodextrin/resveratrol/cisplatin ternary complex; *n* = 6). *p*-level values lower than 0.05 are bolded.

| | *"C"* | *"P"* | *"G"* | *"PG"* | *"RG"* | *"RP"* |
|---|---|---|---|---|---|---|
| *"C"* | | 0.880581 | 0.999755 | 0.997091 | 0.101061 | 0.724562 |
| *"P"* | | | 0.960582 | 0.985953 | **0.010903** | 0.179183 |
| *"G"* | | | | 0.999971 | 0.055328 | 0.552422 |
| *"PG"* | | | | | **0.036561** | 0.441577 |
| *"RG"* | | | | | | 0.772121 |
| *"RP"* | | | | | | |

**Table S8.** Significant *p*-levels from the Tukey HSD test for the GRA (Granulocytes, $10^9$/L) parameter values determined from the Wistar rat blood samples ("C" – control group, "P" – group treated with cisplatin, "G" – group treated with "empty" γ-cyclodextrin, "PG" – group treated with γ-cyclodextrin/cisplatin binary complex, "RG" – group treated with γ-cyclodextrin/resveratrol binary complex, "RP" – group treated with γ-cyclodextrin/resveratrol/cisplatin ternary complex; *n* = 6).

| | *"C"* | *"P"* | *"G"* | *"PG"* | *"RG"* | *"RP"* |
|---|---|---|---|---|---|---|
| *"C"* | | 0.847074 | 0.973531 | 0.885463 | 0.499619 | 0.752879 |
| *"P"* | | | 0.997359 | 0.999992 | 0.995116 | 0.999996 |
| *"G"* | | | | 0.999469 | 0.907732 | 0.989987 |
| *"PG"* | | | | | 0.981678 | 0.999781 |
| *"RG"* | | | | | | 0.998296 |
| *"RP"* | | | | | | |

**Table S9.** Significant *p*-levels from the Tukey HSD test for the LY% (Lymphocytes, %) parameter values determined from the Wistar rat blood samples ("C" – control group, "P" – group treated with cisplatin, "G" – group treated with "empty" γ-cyclodextrin, "PG" – group treated with γ-cyclodextrin/cisplatin binary complex, "RG" – group treated with γ-cyclodextrin/resveratrol binary complex, "RP" – group treated with γ-cyclodextrin/resveratrol/cisplatin ternary complex; *n* = 6).

| | *"C"* | *"P"* | *"G"* | *"PG"* | *"RG"* | *"RP"* |
|---|---|---|---|---|---|---|
| *"C"* | | 0.159879 | 0.962601 | 0.827155 | 0.818857 | 0.999932 |
| *"P"* | | | 0.533385 | 0.763579 | 0.772454 | 0.106505 |
| *"G"* | | | | 0.998607 | 0.998299 | 0.903815 |
| *"PG"* | | | | | 1.000000 | 0.709695 |
| *"RG"* | | | | | | 0.699611 |
| *"RP"* | | | | | | |

**Table S10.** Significant *p*-levels from the Tukey HSD test for the MI% (Middle Cells, %) parameter values determined from the Wistar rat blood samples ("C" – control group, "P" – group treated with cisplatin, "G" – group treated with "empty" γ-cyclodextrin, "PG" – group treated with γ-cyclodextrin/cisplatin binary complex, "RG" – group treated with γ-cyclodextrin/resveratrol binary complex, "RP" – group treated with γ-cyclodextrin/resveratrol/cisplatin ternary complex; *n* = 6).

| | *"C"* | *"P"* | *"G"* | *"PG"* | *"RG"* | *"RP"* |
|---|---|---|---|---|---|---|
| *"C"* | | 1.000000 | 1.000000 | 0.999999 | 0.119590 | 0.973283 |
| *"P"* | | | 1.000000 | 1.000000 | 0.170442 | 0.985270 |
| *"G"* | | | | 0.999999 | 0.119590 | 0.973283 |
| *"PG"* | | | | | 0.144412 | 0.985345 |
| *"RG"* | | | | | | 0.429389 |
| *"RP"* | | | | | | |

**Table S11.** Significant *p*-levels from the Tukey HSD test for the GR% (Granulocytes, %) parameter values determined from the Wistar rat blood samples ("C" – control group, "P" – group treated with cisplatin, "G" – group treated with "empty" γ-cyclodextrin, "PG" – group treated with γ-cyclodextrin/cisplatin binary complex, "RG" – group treated with γ-cyclodextrin/resveratrol binary complex, "RP" – group treated with γ-cyclodextrin/resveratrol/cisplatin ternary complex; $n = 6$).

| | *"C"* | *"P"* | *"G"* | *"PG"* | *"RG"* | *"RP"* |
|---|---|---|---|---|---|---|
| *"C"* | | 0.144744 | 0.958831 | 0.825181 | 0.998237 | 0.996987 |
| *"P"* | | | 0.512438 | 0.736469 | 0.293998 | 0.057281 |
| *"G"* | | | | 0.998838 | 0.998323 | 0.782801 |
| *"PG"* | | | | | 0.965609 | 0.552996 |
| *"RG"* | | | | | | 0.947060 |
| *"RP"* | | | | | | |

**Table S12.** Significant *p*-levels from the Tukey HSD test for the RBC (Red Blood Cells, $10^{12}$/L) parameter values determined from the Wistar rat blood samples ("C" – control group, "P" – group treated with cisplatin, "G" – group treated with "empty" γ-cyclodextrin, "PG" – group treated with γ-cyclodextrin/cisplatin binary complex, "RG" – group treated with γ-cyclodextrin/resveratrol binary complex, "RP" – group treated with γ-cyclodextrin/resveratrol/cisplatin ternary complex; $n = 6$).

| | *"C"* | *"P"* | *"G"* | *"PG"* | *"RG"* | *"RP"* |
|---|---|---|---|---|---|---|
| *"C"* | | 0.848256 | 0.983585 | 0.999613 | 0.999019 | 0.250841 |
| *"P"* | | | 0.475455 | 0.949854 | 0.962689 | 0.924220 |
| *"G"* | | | | 0.920080 | 0.898675 | 0.067426 |
| *"PG"* | | | | | 1.000000 | 0.404093 |
| *"RG"* | | | | | | 0.440220 |
| *"RP"* | | | | | | |

**Table S13.** Significant *p*-levels from the Tukey HSD test for the HGB (Hemoglobin, g/dL) parameter values determined from the Wistar rat blood samples ("C" – control group, "P" – group treated with cisplatin, "G" – group treated with "empty" γ-cyclodextrin, "PG" – group treated with γ-cyclodextrin/cisplatin binary complex, "RG" – group treated with γ-cyclodextrin/resveratrol binary complex, "RP" – group treated with γ-cyclodextrin/resveratrol/cisplatin ternary complex; $n = 6$).

| | *"C"* | *"P"* | *"G"* | *"PG"* | *"RG"* | *"RP"* |
|---|---|---|---|---|---|---|
| *"C"* | | 0.999134 | 0.688988 | 0.846792 | 0.999844 | 0.905859 |
| *"P"* | | | 0.902995 | 0.973096 | 0.999997 | 0.760951 |
| *"G"* | | | | 0.999700 | 0.829660 | 0.160801 |
| *"PG"* | | | | | 0.939552 | 0.270398 |
| *"RG"* | | | | | | 0.792839 |
| *"RP"* | | | | | | |

**Table S14.** Significant *p*-levels from the Tukey HSD test for the HCT (Hematocrit, %) parameter values determined from the Wistar rat blood samples ("C" – control group, "P" – group treated with cisplatin, "G" – group treated with "empty" γ-cyclodextrin, "PG" – group treated with γ-cyclodextrin/cisplatin binary complex, "RG" – group treated with γ-cyclodextrin/resveratrol binary complex, "RP" – group treated with γ-cyclodextrin/resveratrol/cisplatin ternary complex; *n* = 6).

| | *"C"* | *"P"* | *"G"* | *"PG"* | *"RG"* | *"RP"* |
|---|---|---|---|---|---|---|
| *"C"* | | 0.997190 | 0.757082 | 0.978432 | 0.999356 | 0.849031 |
| *"P"* | | | 0.960687 | 0.999921 | 0.999994 | 0.622607 |
| *"G"* | | | | 0.987796 | 0.912764 | 0.153492 |
| *"PG"* | | | | | 0.998985 | 0.432564 |
| *"RG"* | | | | | | 0.661834 |
| *"RP"* | | | | | | |

**Table S15.** Significant *p*-levels from the Tukey HSD test for the MCV (Mean Corpuscular Volume, fL) parameter values determined from the Wistar rat blood samples ("C" – control group, "P" – group treated with cisplatin, "G" – group treated with "empty" γ-cyclodextrin, "PG" – group treated with γ-cyclodextrin/cisplatin binary complex, "RG" – group treated with γ-cyclodextrin/resveratrol binary complex, "RP" – group treated with γ-cyclodextrin/resveratrol/cisplatin ternary complex; *n* = 6).

| | *"C"* | *"P"* | *"G"* | *"PG"* | *"RG"* | *"RP"* |
|---|---|---|---|---|---|---|
| *"C"* | | 0.219145 | 0.909716 | 0.781061 | 0.909716 | 0.698577 |
| *"P"* | | | 0.753710 | 0.886665 | 0.753710 | 0.933022 |
| *"G"* | | | | 0.999715 | 1.000000 | 0.997892 |
| *"PG"* | | | | | 0.999715 | 0.999991 |
| *"RG"* | | | | | | 0.997892 |
| *"RP"* | | | | | | |

**Table S16.** Significant *p*-levels from the Tukey HSD test for the MCH (Mean Corpuscular Hemoglobin, pg) parameter values determined from the Wistar rat blood samples ("C" – control group, "P" – group treated with cisplatin, "G" – group treated with "empty" γ-cyclodextrin, "PG" – group treated with γ-cyclodextrin/cisplatin binary complex, "RG" – group treated with γ-cyclodextrin/resveratrol binary complex, "RP" – group treated with γ-cyclodextrin/resveratrol/cisplatin ternary complex; *n* = 6).

| | *"C"* | *"P"* | *"G"* | *"PG"* | *"RG"* | *"RP"* |
|---|---|---|---|---|---|---|
| *"C"* | | 0.461284 | 0.942739 | 0.431749 | 0.974310 | 0.695973 |
| *"P"* | | | 0.922878 | 1.000000 | 0.868058 | 0.997507 |
| *"G"* | | | | 0.920296 | 0.999986 | 0.993513 |
| *"PG"* | | | | | 0.861407 | 0.997951 |
| *"RG"* | | | | | | 0.979707 |
| *"RP"* | | | | | | |

**Table S17.** Significant *p*-levels from the Tukey HSD test for the MCHC (Mean Corpuscular Hemoglobin Concentration, g/dL) parameter values determined from the Wistar rat blood samples ("C" – control group, "P" – group treated with cisplatin, "G" – group treated with "empty" γ-cyclodextrin, "PG" – group treated with γ-cyclodextrin/cisplatin binary complex, "RG" – group treated with γ-cyclodextrin/resveratrol binary complex, "RP" – group treated with γ-cyclodextrin/resveratrol/cisplatin ternary complex; $n = 6$).

| | *"C"* | *"P"* | *"G"* | *"PG"* | *"RG"* | *"RP"* |
|---|---|---|---|---|---|---|
| *"C"* | | 1.000000 | 0.999686 | 0.931163 | 0.999999 | 0.999686 |
| *"P"* | | | 0.999345 | 0.925864 | 1.000000 | 0.999345 |
| *"G"* | | | | 0.986017 | 0.998836 | 1.000000 |
| *"PG"* | | | | | 0.902636 | 0.986017 |
| *"RG"* | | | | | | 0.998836 |
| *"RP"* | | | | | | |

**Table S18.** Significant *p*-levels from the Tukey HSD test for the RDWc (Red Blood Cell Distribution Width, %) parameter values determined from the Wistar rat blood samples ("C" – control group, "P" – group treated with cisplatin, "G" – group treated with "empty" γ-cyclodextrin, "PG" – group treated with γ-cyclodextrin/cisplatin binary complex, "RG" – group treated with γ-cyclodextrin/resveratrol binary complex, "RP" – group treated with γ-cyclodextrin/resveratrol/cisplatin ternary complex; $n = 6$).

| | *"C"* | *"P"* | *"G"* | *"PG"* | *"RG"* | *"RP"* |
|---|---|---|---|---|---|---|
| *"C"* | | 0.173993 | 0.536233 | 0.509854 | 0.720209 | 0.682004 |
| *"P"* | | | 0.962354 | 0.969713 | 0.877060 | 0.900512 |
| *"G"* | | | | 1.000000 | 0.999665 | 0.999897 |
| *"PG"* | | | | | 0.999359 | 0.999767 |
| *"RG"* | | | | | | 1.000000 |
| *"RP"* | | | | | | |

**Table S19.** Significant *p*-levels from the Tukey HSD test for the PLT (Platelet/Thrombocytes, $10^9$/L) parameter values determined from the Wistar rat blood samples ("C" – control group, "P" – group treated with cisplatin, "G" – group treated with "empty" γ-cyclodextrin, "PG" – group treated with γ-cyclodextrin/cisplatin binary complex, "RG" – group treated with γ-cyclodextrin/resveratrol binary complex, "RP" – group treated with γ-cyclodextrin/resveratrol/cisplatin ternary complex; $n = 6$).

| | *"C"* | *"P"* | *"G"* | *"PG"* | *"RG"* | *"RP"* |
|---|---|---|---|---|---|---|
| *"C"* | | 0.956895 | 0.999955 | 1.000000 | 0.282822 | 0.939275 |
| *"P"* | | | 0.985779 | 0.964932 | 0.821201 | 1.000000 |
| *"G"* | | | | 0.999986 | 0.383698 | 0.978396 |
| *"PG"* | | | | | 0.302732 | 0.949913 |
| *"RG"* | | | | | | 0.807567 |
| *"RP"* | | | | | | |

**Table S20.** Significant *p*-levels from the Tukey HSD test for the PCT (Plateletcrit, %) parameter values determined from the Wistar rat blood samples ("C" – control group, "P" – group treated with cisplatin, "G" – group treated with "empty" γ-cyclodextrin, "PG" – group treated with γ-cyclodextrin/cisplatin binary complex, "RG" – group treated with γ-cyclodextrin/resveratrol binary complex, "RP" – group treated with γ-cyclodextrin/resveratrol/cisplatin ternary complex; *n* = 6).

| | *"C"* | *"P"* | *"G"* | *"PG"* | *"RG"* | *"RP"* |
|---|---|---|---|---|---|---|
| *"C"* | | 0.948237 | 1.000000 | 0.999999 | 0.177259 | 0.922373 |
| *"P"* | | | 0.962607 | 0.924078 | 0.701740 | 1.000000 |
| *"G"* | | | | 0.999984 | 0.201274 | 0.942155 |
| *"PG"* | | | | | 0.148759 | 0.890256 |
| *"RG"* | | | | | | 0.690151 |
| *"RP"* | | | | | | |

**Table S21.** Significant *p*-levels from the Tukey HSD test for the MPV (Mean Platelet Volume, fL) parameter values determined from the Wistar rat blood samples ("C" – control group, "P" – group treated with cisplatin, "G" – group treated with "empty" γ-cyclodextrin, "PG" – group treated with γ-cyclodextrin/cisplatin binary complex, "RG" – group treated with γ-cyclodextrin/resveratrol binary complex, "RP" – group treated with γ-cyclodextrin/resveratrol/cisplatin ternary complex; *n* = 6).

| | *"C"* | *"P"* | *"G"* | *"PG"* | *"RG"* | *"RP"* |
|---|---|---|---|---|---|---|
| *"C"* | | 0.999944 | 0.971500 | 0.880995 | 0.715966 | 1.000000 |
| *"P"* | | | 0.933692 | 0.809544 | 0.858706 | 0.999944 |
| *"G"* | | | | 0.999482 | 0.276434 | 0.971500 |
| *"PG"* | | | | | 0.154469 | 0.880995 |
| *"RG"* | | | | | | 0.715966 |
| *"RP"* | | | | | | |

**Table S22.** Significant *p*-levels from the Tukey HSD test for the PDWc (Platelet Distribution Width, %) parameter values determined from the Wistar rat blood samples ("C" – control group, "P" – group treated with cisplatin, "G" – group treated with "empty" γ-cyclodextrin, "PG" – group treated with γ-cyclodextrin/cisplatin binary complex, "RG" – group treated with γ-cyclodextrin/resveratrol binary complex, "RP" – group treated with γ-cyclodextrin/resveratrol/cisplatin ternary complex; *n* = 6).

| | *"C"* | *"P"* | *"G"* | *"PG"* | *"RG"* | *"RP"* |
|---|---|---|---|---|---|---|
| *"C"* | | 0.999996 | 0.525084 | 0.680366 | 0.794493 | 0.988153 |
| *"P"* | | | 0.501135 | 0.649414 | 0.877925 | 0.978400 |
| *"G"* | | | | 0.999860 | 0.055058 | 0.876495 |
| *"PG"* | | | | | 0.094507 | 0.954846 |
| *"RG"* | | | | | | 0.420168 |
| *"RP"* | | | | | | |

### 3. Tukey HSD tests for the biochemical parameters

**Table S23.** Significant *p*-levels from the Tukey HSD test for the GOT/AST (glutamic-oxalacetic transaminase / aspartate transaminase, expressed as IU/L) parameter values determined from the Wistar rat plasma samples ("C" – control group, "PG" – group treated with $\gamma$-cyclodextrin/cisplatin binary complex, "RP" – group treated with $\gamma$-cyclodextrin/resveratrol/cisplatin ternary complex; $n = 6$).

| | *"C"* | *"PG"* | *"RP"* |
|---|---|---|---|
| *"C"* | | 0.322302 | 0.932781 |
| *"PG"* | | | 0.188035 |
| *"RP"* | | | |

**Table S24.** Significant *p*-levels from the Tukey HSD test for the GPT/ALT (glutamic-pyruvic transaminase/ alanine transaminase, IU/L) parameter values determined from the Wistar rat plasma samples ("C" – control group, "PG" – group treated with $\gamma$-cyclodextrin/cisplatin binary complex, "RP" – group treated with $\gamma$-cyclodextrin/resveratrol/cisplatin ternary complex; $n = 6$).

| | *"C"* | *"PG"* | *"RP"* |
|---|---|---|---|
| *"C"* | | 0.329630 | 0.871814 |
| *"PG"* | | | 0.603269 |
| *"RP"* | | | |

**Table S25.** Significant *p*-levels from the Tukey HSD test for the GGT (gamma-glutamyl transferase, IU/L) parameter values determined from the Wistar rat plasma samples ("C" – control group, "PG" – group treated with $\gamma$-cyclodextrin/cisplatin binary complex, "RP" – group treated with $\gamma$-cyclodextrin/resveratrol/cisplatin ternary complex; $n = 6$).

| | *"C"* | *"PG"* | *"RP"* |
|---|---|---|---|
| *"C"* | | 0.746587 | 0.620738 |
| *"PG"* | | | 0.244274 |
| *"RP"* | | | |

**Table S26.** Significant *p*-levels from the Tukey HSD test for the T-Bil (Total Bilirubin, mg/dL) parameter values determined from the Wistar rat plasma samples ("C" – control group, "PG" – group treated with $\gamma$-cyclodextrin/cisplatin binary complex, "RP" – group treated with $\gamma$-cyclodextrin/resveratrol/cisplatin ternary complex; $n = 6$).

| | *"C"* | *"PG"* | *"RP"* |
|---|---|---|---|
| *"C"* | | 0.210438 | 0.944313 |
| *"PG"* | | | 0.339567 |
| *"RP"* | | | |

**Table S27.** Significant *p*-levels from the Tukey HSD test for the BUN (Blood Urea Nitrogen, mg/dL) parameter values determined from the Wistar rat plasma samples ("C" – control group, "PG" – group treated with γ-cyclodextrin/cisplatin binary complex, "RP" – group treated with γ-cyclodextrin/resveratrol/cisplatin ternary complex; *n* = 6). *p*-level values lower than 0.05 are bolded.

| | *"C"* | *"PG"* | *"RP"* |
|---|---|---|---|
| *"C"* | | **0.011639** | 0.396263 |
| *"PG"* | | | 0.144240 |
| *"RP"* | | | |

**Table S28.** Significant *p*-levels from the Tukey HSD test for the UA (Uric Acid, mg/dL) parameter values determined from the Wistar rat plasma samples ("C" – control group, "PG" – group treated with γ-cyclodextrin/cisplatin binary complex, "RP" – group treated with γ-cyclodextrin/resveratrol/cisplatin ternary complex; *n* = 6). *p*-level values lower than 0.05 are bolded.

| | *"C"* | *"PG"* | *"RP"* |
|---|---|---|---|
| *"C"* | | **0.000178** | 0.838998 |
| *"PG"* | | | **0.000178** |
| *"RP"* | | | |

**Table S29.** Significant *p*-levels from the Tukey HSD test for the Cre (Creatinine, mg/dL) parameter values determined from the Wistar rat plasma samples ("C" – control group, "PG" – group treated with γ-cyclodextrin/cisplatin binary complex, "RP" – group treated with γ-cyclodextrin/resveratrol/cisplatin ternary complex; *n* = 6). *p*-level values lower than 0.05 are bolded.

| | *"C"* | *"PG"* | *"RP"* |
|---|---|---|---|
| *"C"* | | **0.000622** | 0.594743 |
| *"PG"* | | | **0.003526** |
| *"RP"* | | | |

**Table S30.** Significant *p*-levels from the Tukey HSD test for the T-Pro (Total Proteins, g/dL) parameter values determined from the Wistar rat plasma samples ("C" – control group, "PG" – group treated with γ-cyclodextrin/cisplatin binary complex, "RP" – group treated with γ-cyclodextrin/resveratrol/cisplatin ternary complex; *n* = 6). *p*-level values lower than 0.05 are bolded.

| | *"C"* | *"PG"* | *"RP"* |
|---|---|---|---|
| *"C"* | | **0.004647** | 0.142479 |
| *"PG"* | | | 0.203795 |
| *"RP"* | | | |

**Table S31.** Significant *p*-levels from the Tukey HSD test for the Alb (Albumins, g/dL) parameter values determined from the Wistar rat plasma samples ("C" – control group, "PG" – group treated with γ-cyclodextrin/cisplatin binary complex, "RP" – group treated with γ-cyclodextrin/resveratrol/cisplatin ternary complex; *n* = 6). *p*-level values lower than 0.05 are bolded.

| | *"C"* | *"PG"* | *"RP"* |
|---|---|---|---|
| *"C"* | | **0.005319** | **0.025386** |
| *"PG"* | | | 0.711974 |
| *"RP"* | | | |

**Table S32.** Significant *p*-levels from the Tukey HSD test for the ALP (Alkaline Phosphatase, IU/L) parameter values determined from the Wistar rat plasma samples ("C" – control group, "PG" – group treated with γ-cyclodextrin/cisplatin binary complex, "RP" – group treated with γ-cyclodextrin/resveratrol/cisplatin ternary complex; *n* = 6).

| | *"C"* | *"PG"* | *"RP"* |
|---|---|---|---|
| *"C"* | | 0.378597 | 0.765366 |
| *"PG"* | | | 0.783569 |
| *"RP"* | | | |

**Table S33.** Significant *p*-levels from the Tukey HSD test for the CPK (Creatine Phosphokinase, IU/L) parameter values determined from the Wistar rat plasma samples ("C" – control group, "PG" – group treated with γ-cyclodextrin/cisplatin binary complex, "RP" – group treated with γ-cyclodextrin/resveratrol/cisplatin ternary complex; *n* = 6). *p*-level values lower than 0.05 are bolded.

| | *"C"* | *"PG"* | *"RP"* |
|---|---|---|---|
| *"C"* | | **0.004863** | 0.593253 |
| *"PG"* | | | **0.034050** |
| *"RP"* | | | |

**Table S34.** Significant *p*-levels from the Tukey HSD test for the Calcium (Ca, mg/dL) parameter values determined from the Wistar rat plasma samples ("C" – control group, "PG" – group treated with γ-cyclodextrin/cisplatin binary complex, "RP" – group treated with γ-cyclodextrin/resveratrol/cisplatin ternary complex; *n* = 6).

| | *"C"* | *"PG"* | *"RP"* |
|---|---|---|---|
| *"C"* | | 0.142544 | 1.000000 |
| *"PG"* | | | 0.142544 |
| *"RP"* | | | |

**Table S35.** Significant *p*-levels from the Tukey HSD test for the Magnesium (Mg, mg/dL) parameter values determined from the Wistar rat plasma samples ("C" – control group, "PG" – group treated with γ-cyclodextrin/cisplatin binary complex, "RP" – group treated with γ-cyclodextrin/resveratrol/cisplatin ternary complex; *n* = 6). *p*-level values lower than 0.05 are bolded.

| | *"C"* | *"PG"* | *"RP"* |
|---|---|---|---|
| *"C"* | | **0.001429** | 0.761475 |
| *"PG"* | | | **0.000471** |
| *"RP"* | | | |

**Table S36.** Significant *p*-levels from the Tukey HSD test for the Inorganic phosphorus (IP, mg/dL) parameter values determined from the Wistar rat plasma samples ("C" – control group, "PG" – group treated with γ-cyclodextrin/cisplatin binary complex, "RP" – group treated with γ-cyclodextrin/resveratrol/cisplatin ternary complex; *n* = 6). *p*-level values lower than 0.05 are bolded.

| | *"C"* | *"PG"* | *"RP"* |
|---|---|---|---|
| *"C"* | | 0.617583 | 0.113953 |
| *"PG"* | | | **0.019069** |
| *"RP"* | | | |

**4. Principal component analysis of the hematological parameters**

The attempt to analyze the data from all hematological parameter determinations using the PCA multivariate statistical analysis technique do not provides valuable classifications. Almost 100% of the variance was explained by $PC_1$, by involving PLT as the main variable (Figures S1 and S2 for the $PC_2$ versus $PC_1$ scores and loadings plots, respectively). The next step was the removing of the PLT, GR% and LY% variables for the PCA analysis. The first two components explained 63% of the variance of the data, with 48% for the $PC_1$. Most of the antioxidant-protected γ-CD/Rv/CP ternary complex based group was located in the center-left side of the $PC_2$ versus $PC_1$ scores plot, but without a clear classification. However, the "P" and non-antioxidant protected "PG" groups were especially located in the right and upper side of this plot. WBC and LYM had the highest influence for the $PC_1$ (positive side), while RDWc and MCV mostly influenced the classification for the positive and negative sides of the $PC_2$, respectively (Figures S3 and S4).

The above-mentioned results suggest the comparison of non-antioxidant protected and antioxidant protected treatment groups, as well as the control group ("PG", "RP" and "C", respectively). In this PCA analysis, LYM, MID, GRA, RBC, HGB, HCT, MCH, MCHC, RDWc, PCT, MPV and PDWc parameters were used. The non-antioxidant protected γ-CD/CP binary complex treated group was located in the center-top region and the antioxidant protected "RP" ternary complex cases were especially observed in the center-left side of the $PC_2$ versus $PC_1$ scores plot. Most of the control "C" cases were distributed along the $PC_1$ axis. These classification was obtained due to the data variance for LYM, HCT, GRA and RDWc for $PC_1$, and HCT, GRA, RDWc and PDWc for $PC_2$, according to the corresponding loadings plot (Figures S5 and S6).

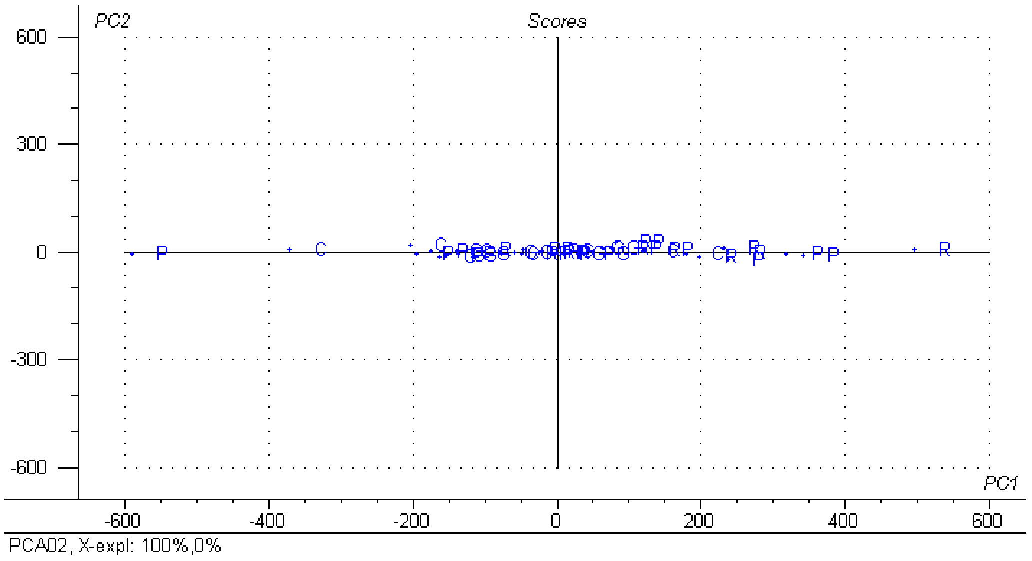


**Figure S1.** $PC_2$ versus $PC_1$ scores plot from the PCA analysis of the hematological parameters determined from the Wistar rat blood samples ("C" – control group, "P" – group treated with cisplatin, "G" – group treated with "empty" γ-cyclodextrin, "PG" – group treated with γ-cyclodextrin/cisplatin binary complex, "RG" – group treated with γ-cyclodextrin/resveratrol binary complex, "RP" – group treated with γ-cyclodextrin/resveratrol/cisplatin ternary complex).

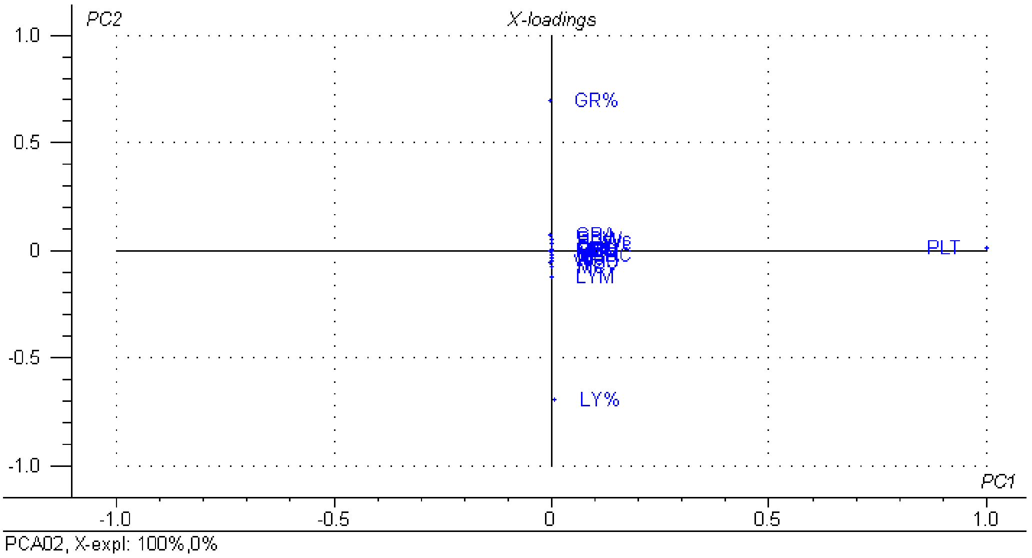


**Figure S2.** $PC_2$ versus $PC_1$ loadings plot from the PCA analysis of the hematological parameters determined from the Wistar rat blood samples.

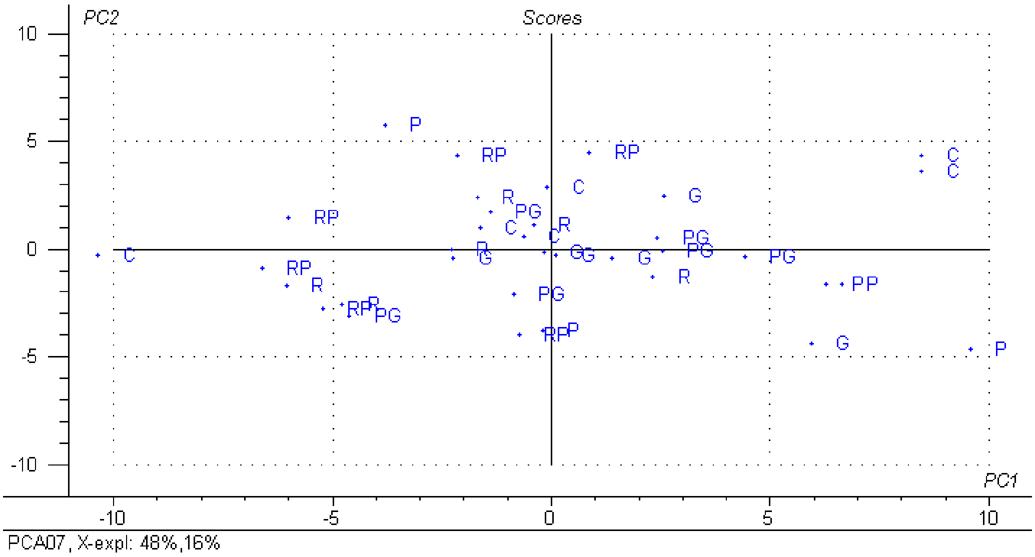


**Figure S3.** $PC_2$ versus $PC_1$ scores plot from the PCA analysis of the hematological parameters (excepting PLT, GR% and LY%) determined from the Wistar rat blood samples ("C" – control group, "P" – group treated with cisplatin, "G" – group treated with "empty" γ-cyclodextrin, "PG" – group treated with γ-cyclodextrin/cisplatin binary complex, "RG" – group treated with γ-cyclodextrin/resveratrol binary complex, "RP" – group treated with γ-cyclodextrin/resveratrol/cisplatin ternary complex).

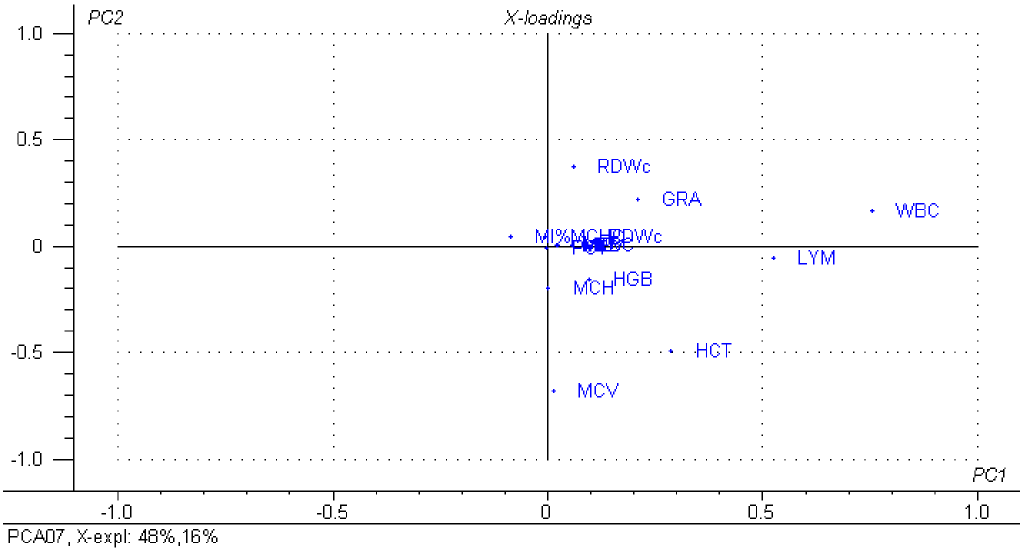


**Figure S4.** $PC_2$ versus $PC_1$ loadings plot from the PCA analysis of the hematological parameters (excepting PLT, GR% and LY%) determined from the Wistar rat blood samples.

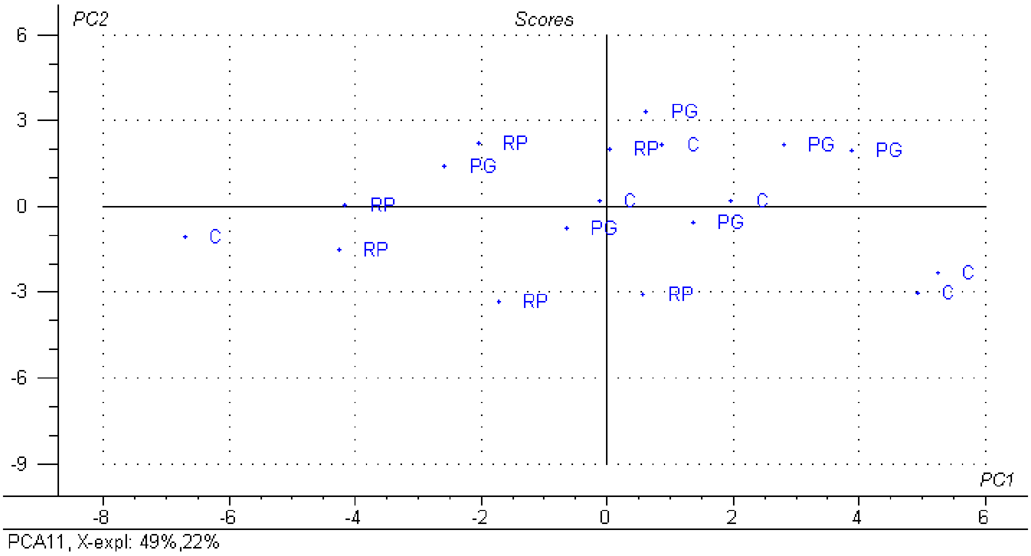


**Figure S5.** $PC_2$ versus $PC_1$ scores plot from the PCA analysis of the hematological parameters (only LYM, MID, GRA, RBC, HGB, HCT, MCH, MCHC, RDWc, PCT, MPV and PDWc) determined from the Wistar rat blood samples (only "C" – control group, "PG" – group treated with γ-cyclodextrin/cisplatin binary complex and "RP" – group treated with γ-cyclodextrin/resveratrol/cisplatin ternary complex).

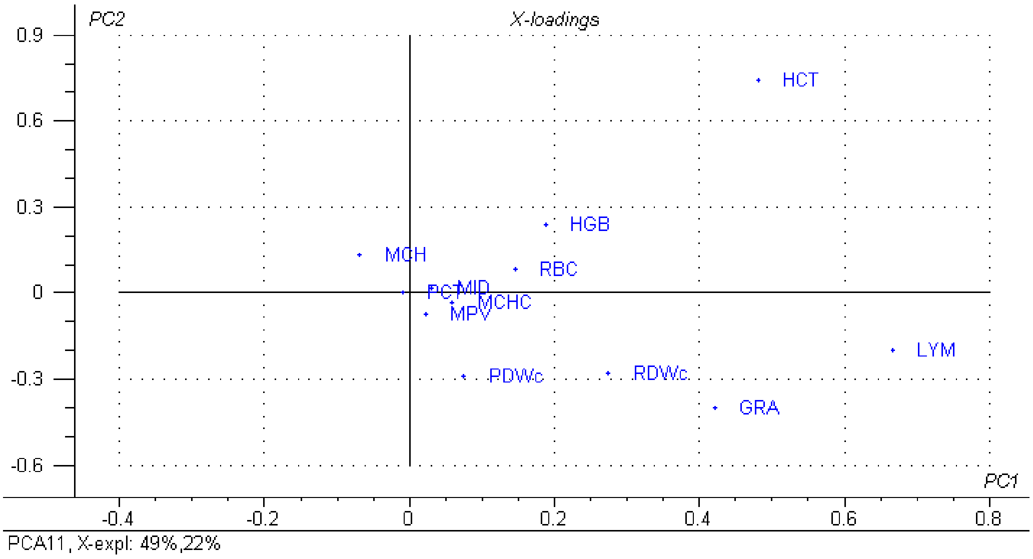


**Figure S6.** $PC_2$ versus $PC_1$ loadings plot from the PCA analysis of the hematological parameters (only LYM, MID, GRA, RBC, HGB, HCT, MCH, MCHC, RDWc, PCT, MPV and PDWc) determined from the Wistar rat blood samples (only "C" – control group, "PG" – group treated with γ-cyclodextrin/cisplatin binary complex and "RP" – group treated with γ-cyclodextrin/resveratrol/cisplatin ternary complex).

**5. Principal component analysis of the biochemical parameters**

Similar PCA approach was performed for the biochemical parameter data. If all biochemical parameters were used as input variables for the PCA analysis, no clear classification was obtained. The distribution of cases is enhanced by removing the ALP and CPK variables. The $PC_2$ versus $PC_1$ scores plot shows a distribution of the "PG" cases in the center-right region, while the antioxidant-protected cases and control group are especially located in the center-left side. However, the classification of the studied groups is not appropriate. Not even if the ALP, CPK, GOT/AST and GPT/ALT variables are removed, it is not obtained better results (Figures S7-S12). The attempt to use all hematological and biochemical parameters as input variables in the PCA analysis did not improve the classification (Figures S13 and S14).

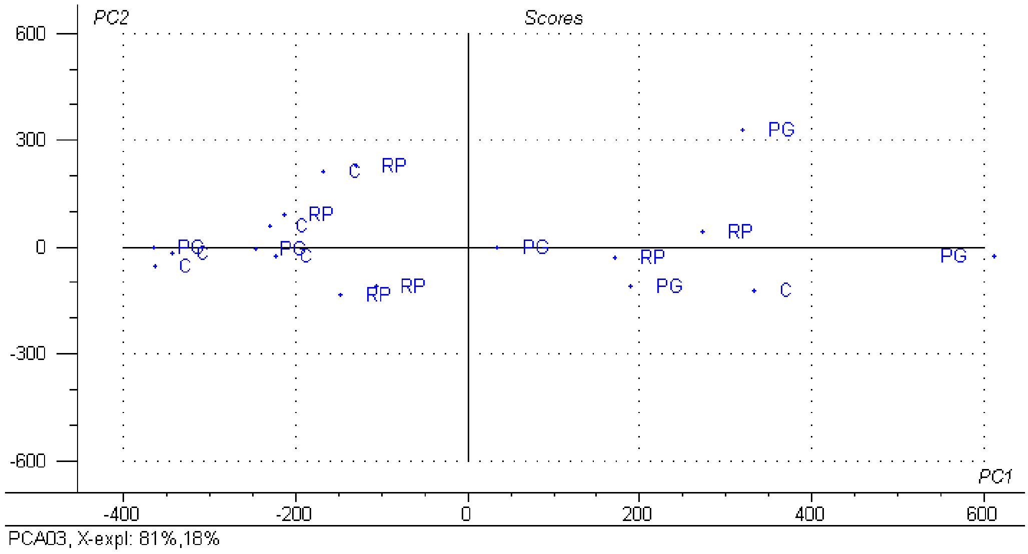


**Figure S7.** $PC_2$ versus $PC_1$ scores plot from the PCA analysis of all biochemical parameters determined from the Wistar rat plasma samples ("C" – control group, "PG" – group treated with γ-cyclodextrin/cisplatin binary complex, "RP" – group treated with γ-cyclodextrin/resveratrol/cisplatin ternary complex).

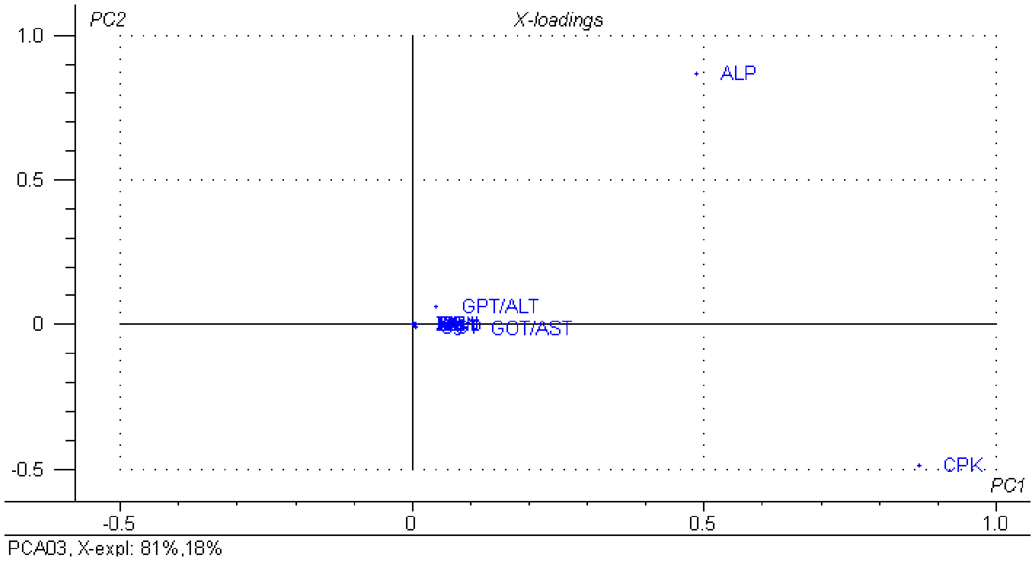


**Figure S8.** $PC_2$ versus $PC_1$ loadings plot from the PCA analysis of all biochemical parameters determined from the Wistar rat plasma samples.

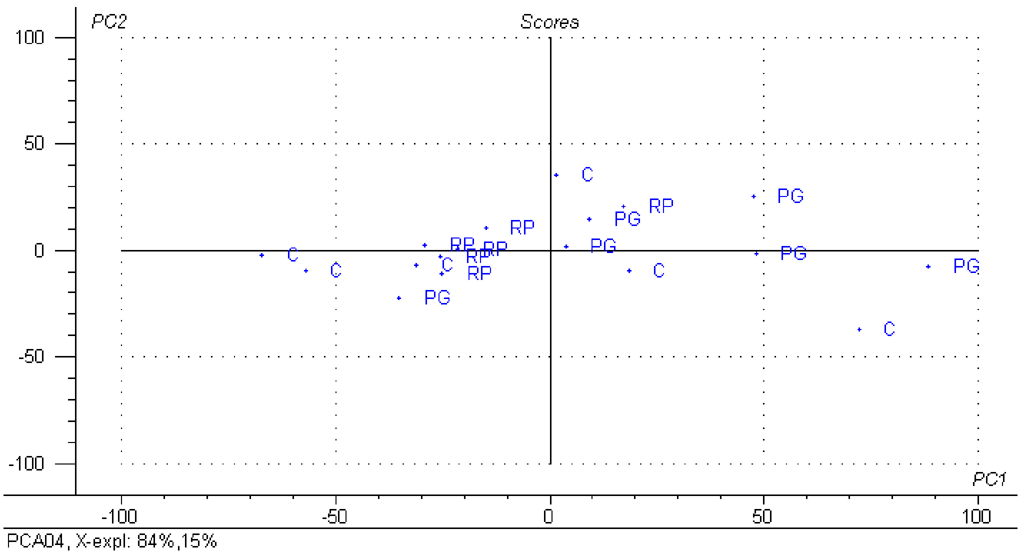


**Figure S9.** PC2 versus PC1 scores plot from the PCA analysis of biochemical parameters (excepting ALP and CPK) determined from the Wistar rat plasma samples ("C" – control group, "PG" – group treated with γ-cyclodextrin/cisplatin binary complex, "RP" – group treated with γ-cyclodextrin/resveratrol/cisplatin ternary complex).

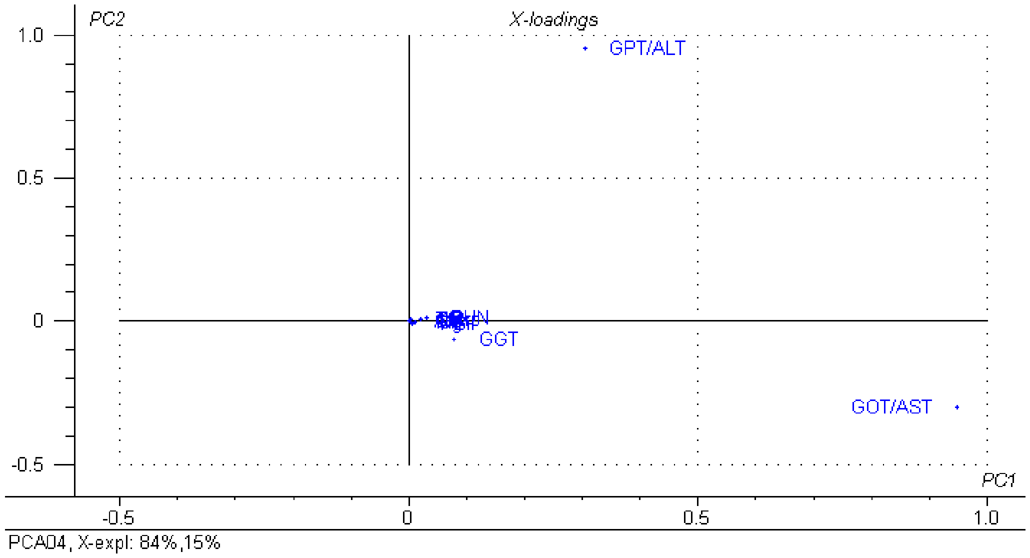


**Figure S10.** PC2 versus PC1 loadings plot from the PCA analysis of biochemical parameters (excepting ALP and CPK) determined from the Wistar rat plasma samples.

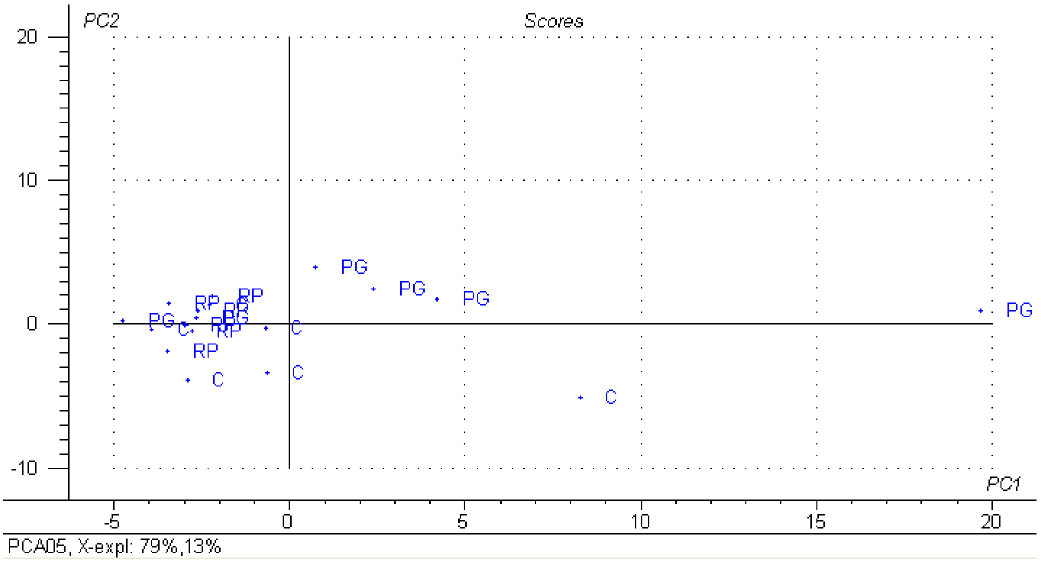


**Figure S11.** PC2 versus PC1 scores plot from the PCA analysis of biochemical parameters (excepting ALP, CPK, GOT/AST and GPT/ALT) determined from the Wistar rat plasma samples ("C" – control group, "PG" – group treated with γ-cyclodextrin/cisplatin binary complex, "RP" – group treated with γ-cyclodextrin/resveratrol/cisplatin ternary complex).

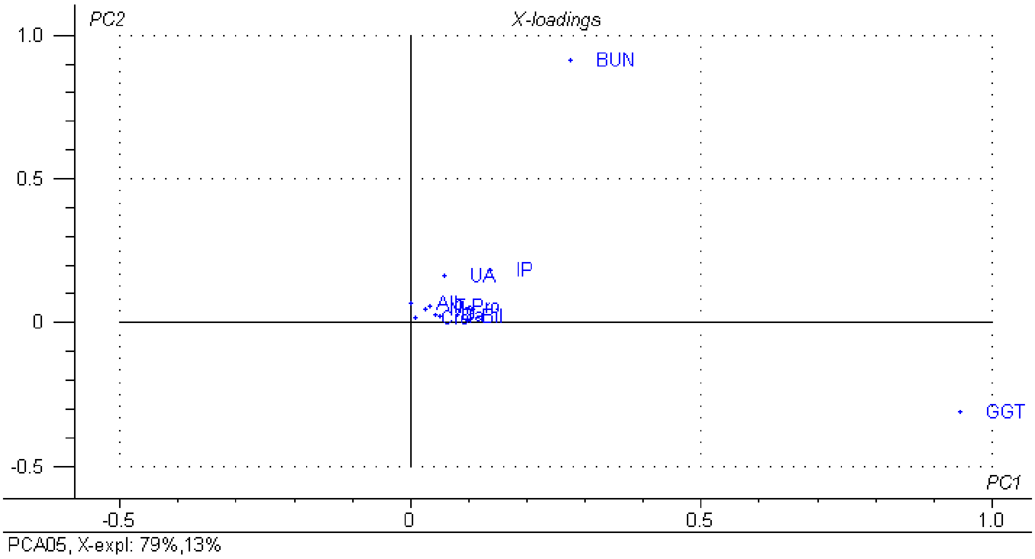


**Figure S12.** PC2 versus PC1 loadings plot from the PCA analysis of biochemical parameters (excepting ALP, CPK, GOT/AST and GPT/ALT) determined from the Wistar rat plasma samples.

**6. Principal component analysis of both hematological and biochemical parameters**

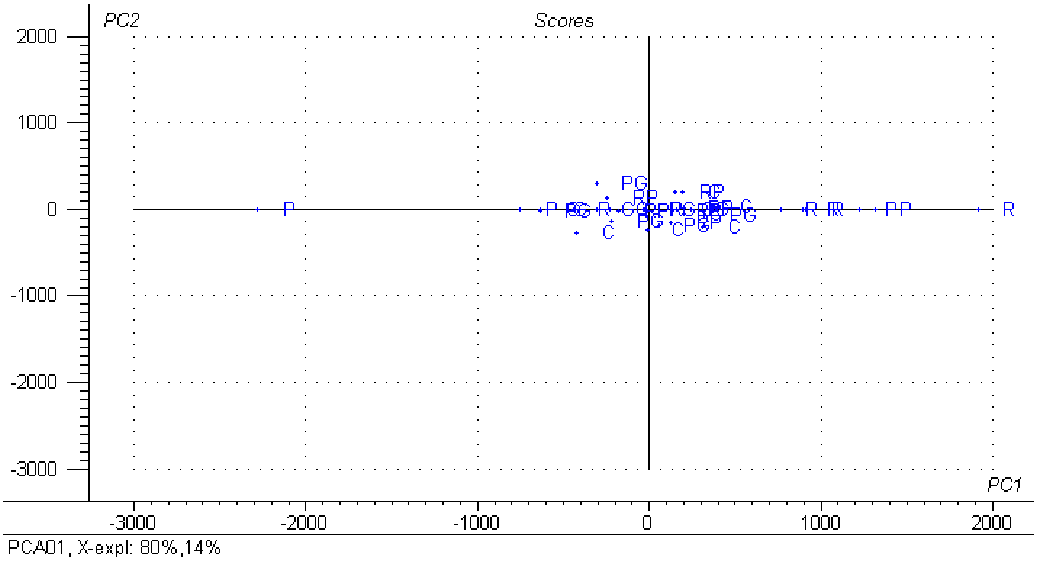


**Figure S13.** PC2 versus PC1 scores plot from the PCA analysis of all hematological and biochemical parameters determined from the Wistar rat blood or plasma samples ("C" – control group, "P" – group treated with cisplatin, "G" – group treated with "empty" γ-cyclodextrin, "PG" – group treated with γ-cyclodextrin/cisplatin binary complex, "RG" – group treated with γ-cyclodextrin/resveratrol binary complex, "RP" – group treated with γ-cyclodextrin/resveratrol/cisplatin ternary complex).

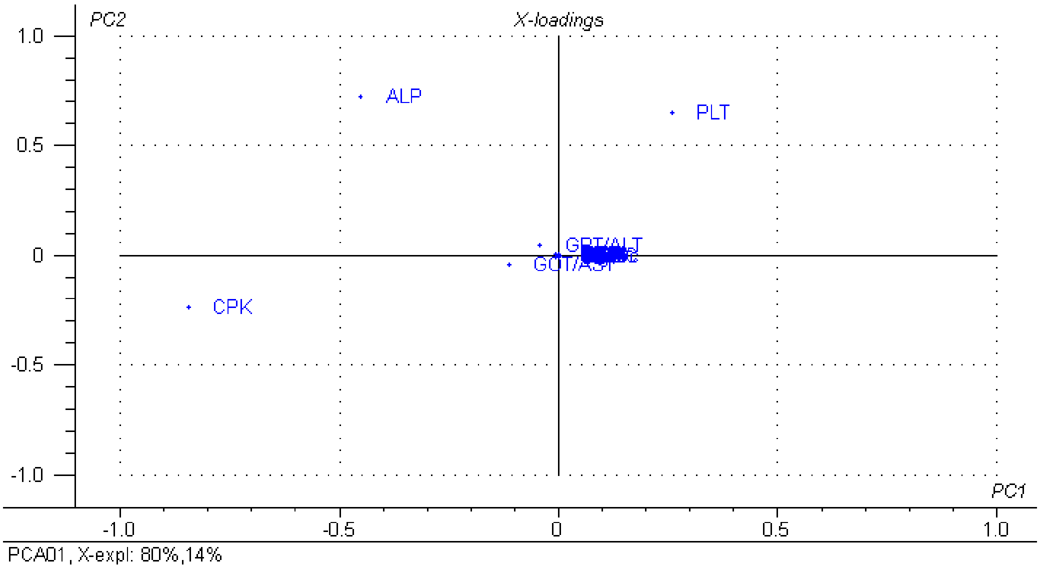


**Figure S14.** PC2 versus PC1 loadings plot from the PCA analysis of all hematological and biochemical parameters determined from the Wistar rat blood or plasma samples ("C" – control group, "P" – group treated with cisplatin, "G" – group treated with "empty" γ-cyclodextrin, "PG" – group treated with γ-cyclodextrin/cisplatin binary complex, "RG" – group treated with γ-cyclodextrin/resveratrol binary complex, "RP" – group treated with γ-cyclodextrin/resveratrol/cisplatin ternary complex).

**7. Principal component analysis of both the kidney-related and element-based biochemical parameters**

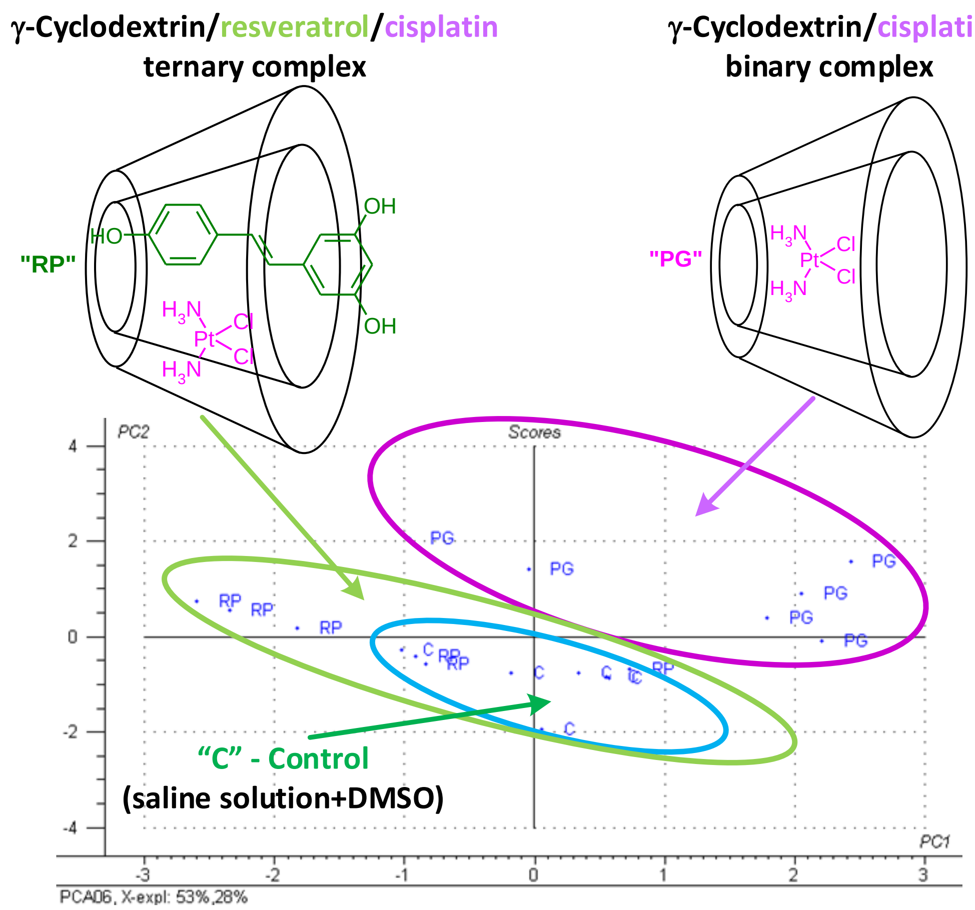


**Figure S15.** PC2 versus PC1 scores plot from the PCA analysis of the significant biochemical parameters (T-Bil, UA, Cre, T-Pro, Alb, Ca, Mg and IP) determined from the Wistar rat plasma samples ("C" – control group, "PG" – group treated with γ-cyclodextrin/cisplatin binary complex, "RP" – group treated with γ-cyclodextrin/resveratrol/cisplatin ternary complex).

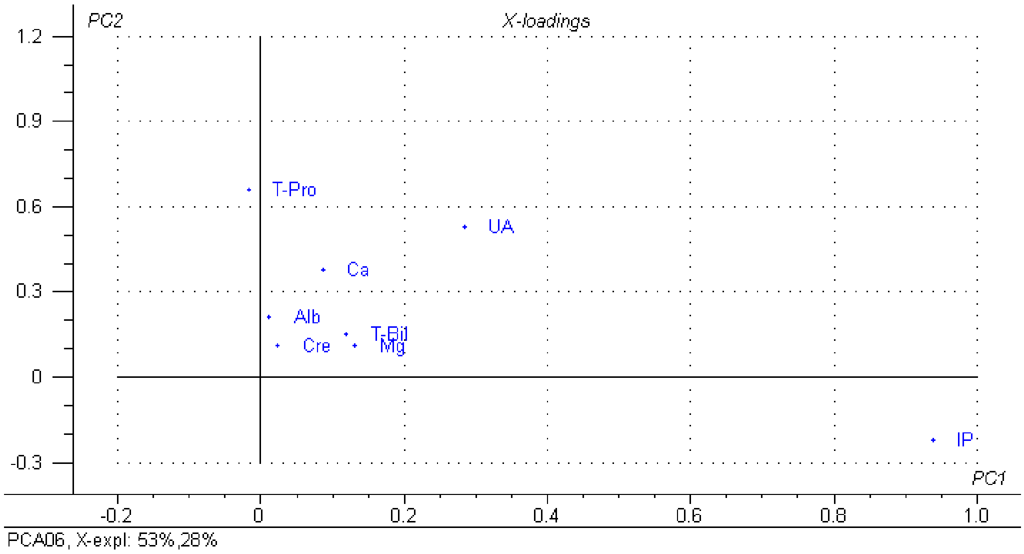


**Figure S16.** PC2 versus PC1 loadings plot from the PCA analysis of the significant biochemical parameters (T-Bil, UA, Cre, T-Pro, Alb, Ca, Mg and IP) determined from the Wistar rat plasma samples.

## 8. Molecular modeling and docking experiments

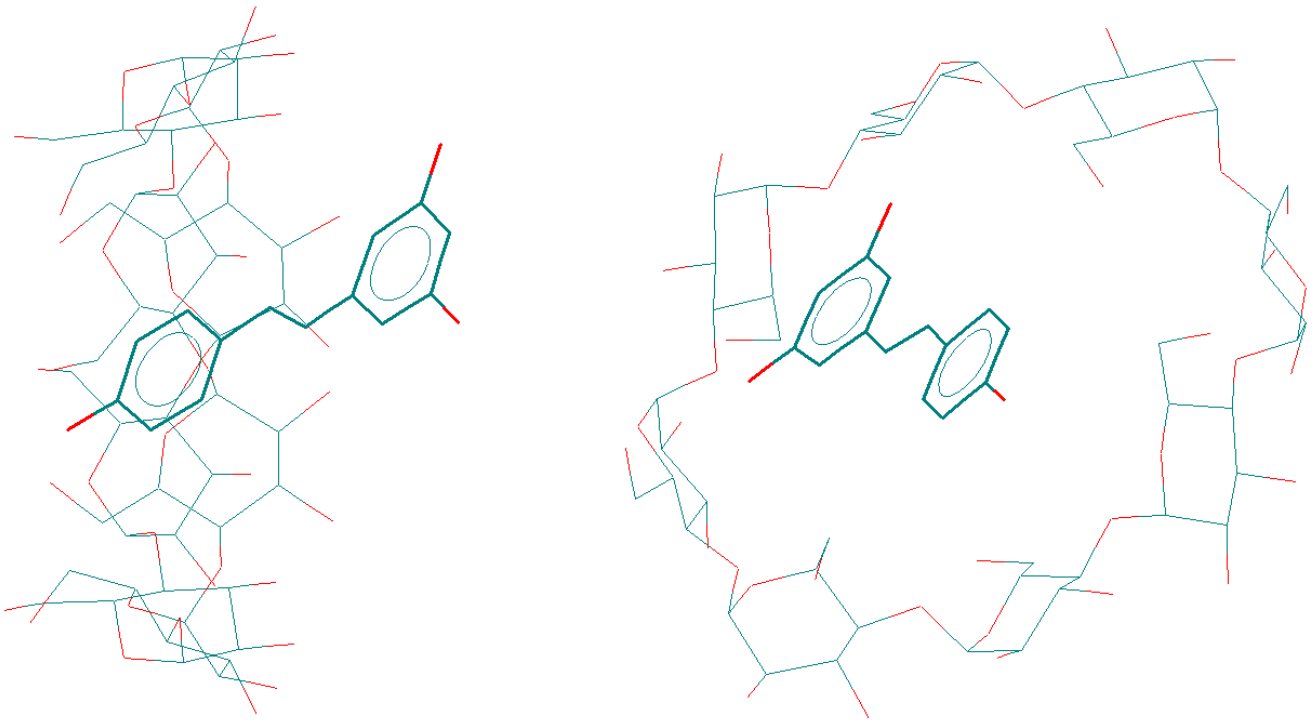

**Figure S17.** Molecular modeled γ-cyclodextrin/resveratrol binary complex at the minimum energy stage, obtained by MM+ geometry optimization; guest compound is bolded.

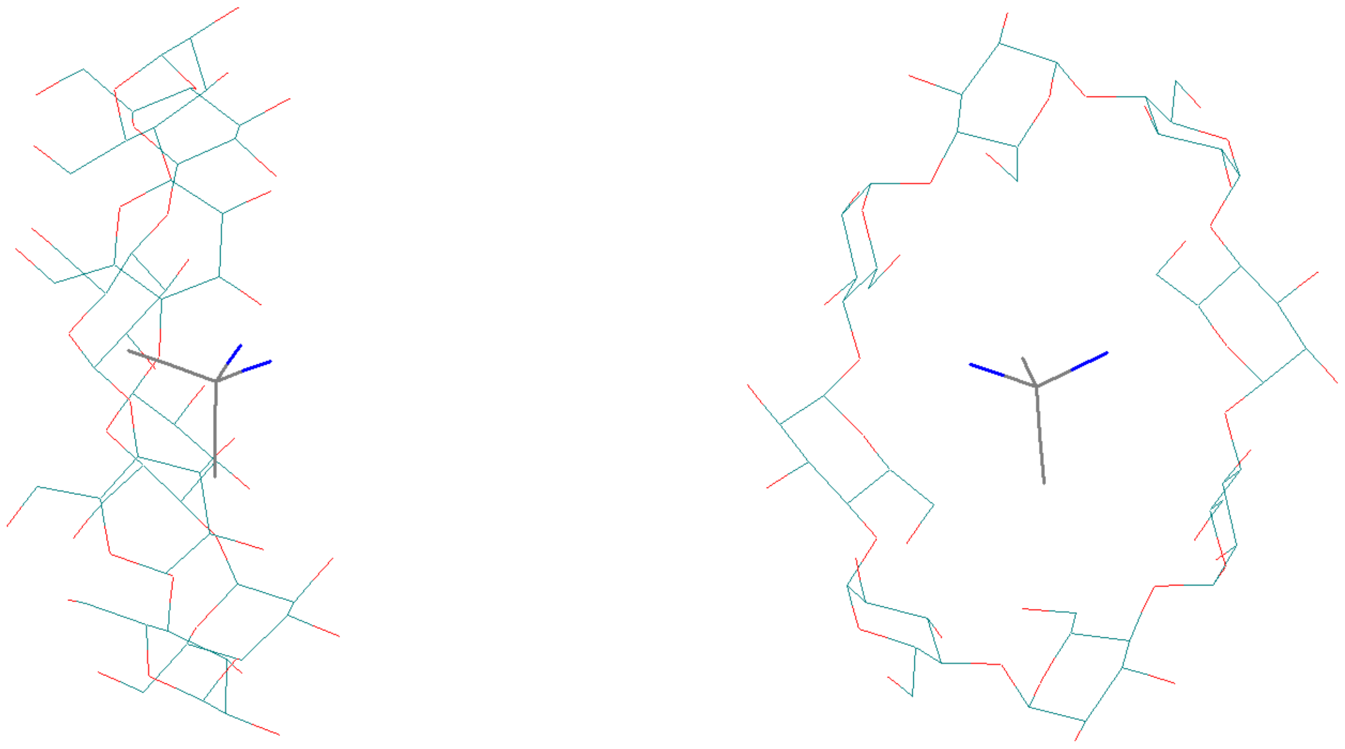

**Figure S18.** Molecular modeled γ-cyclodextrin/cisplatin binary complex at the minimum energy stage, obtained by MM+ geometry optimization; guest compound is bolded.
